# Emerging Therapeutic Potential of SIRT6 Modulators

**DOI:** 10.1021/acs.jmedchem.1c00601

**Published:** 2021-07-02

**Authors:** Francesco Fiorentino, Antonello Mai, Dante Rotili

**Affiliations:** † Department of Chemistry, University of Oxford, South Parks Road, Oxford OX1 3QZ, United Kingdom; ‡ Department of Drug Chemistry & Technologies, Sapienza University of Rome, P.le A Moro 5, 00185 Rome, Italy

## Abstract

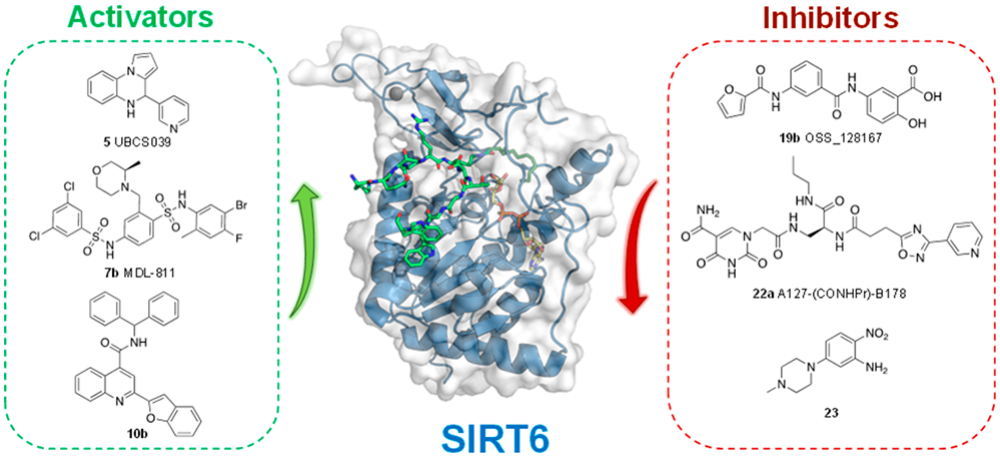

Sirtuin 6 (SIRT6) is an NAD^+^-dependent protein deacylase and mono-ADP-ribosyltransferase of the sirtuin family with a wide substrate specificity. *In vitro* and *in vivo* studies have indicated that SIRT6 overexpression or activation has beneficial effects for cellular processes such as DNA repair, metabolic regulation, and aging. On the other hand, SIRT6 has contrasting roles in cancer, acting either as a tumor suppressor or promoter in a context-specific manner. Given its central role in cellular homeostasis, SIRT6 has emerged as a promising target for the development of small-molecule activators and inhibitors possessing a therapeutic potential in diseases ranging from cancer to age-related disorders. Moreover, specific modulators allow the molecular details of SIRT6 activity to be scrutinized and further validate the enzyme as a pharmacological target. In this Perspective, we summarize the current knowledge about SIRT6 pharmacology and medicinal chemistry and describe the features of the activators and inhibitors identified so far.

## Introduction

**Figure 1:**
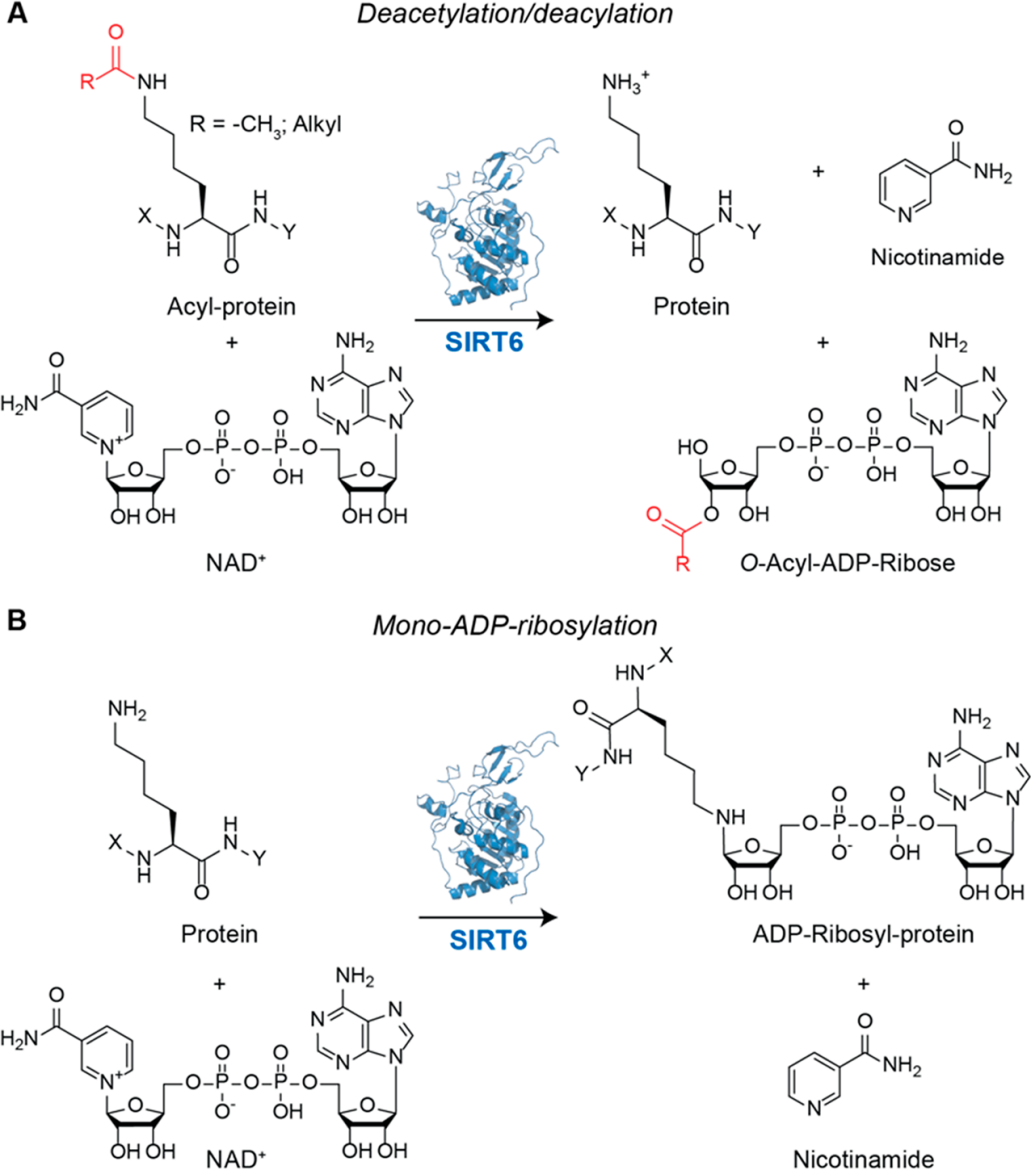
(A) Deacetylation/deacylation reaction catalyzed by SIRT6. The acetyl/acyl group is transferred to an NAD^+^ acceptor, coupled with removal of nicotinamide. (B) Mono-ADP-ribosylation reaction. In this case, ADP–ribose is transferred onto the ε-amino group of lysine from an NAD^+^ donor. Nicotinamide and ADP–ribosyl protein are the products.

The sirtuin family is a class of enzymes that employs NAD^+^ as cofactor.^1^ Although initially classified as class III HDACs, sirtuins (SIRTs) are capable of catalyzing different reactions and possess a wide range of substrates far beyond histones.^2^ Among them, sirtuin 6 (SIRT6) is a pivotal chromatin homeostasis modulator that deacetylates both histone and nonhistone proteins, including DNA repair factors and glucose homeostasis regulators. In addition, SIRT6 promotes the deacylation of long-chain fatty-acid groups and catalyzes the mono-ADP-ribosylation of chromatin silencing DNA repair proteins,^3^ including self-mono-ADP-ribosylation.^4^ Through its enzymatic activity, SIRT6 facilitates the removal of acyl groups from the ε-amino group of lysines and transfers ADP–ribose moieties to lysine and arginine residues of protein substrates (Figure 1).

Given the requirement of NAD^+^ for their activity, SIRTs have been regarded as pivotal proteins connecting metabolism to cellular physiology.^5^ SIRT6, being a nuclear member of this family, tightly regulates DNA repair and genome maintenance and has a pivotal role in glucose and lipid metabolism. These activities are tightly related to the central roles that SIRT6 has in aging, stem cell differentiation, and tumorigenesis.

Loss-of-function studies performed in mouse models indicated the crucial roles that SIRT6 plays for organism wellbeing. Indeed, SIRT6-deficient mice displayed alteration of glycolysis and genomic instability, ultimately leading to premature aging and shortened lifespan.^6−8^ In addition, SIRT6 deletion was associated with increased tumor aggressiveness, and later studies in human cancers identified mutations impairing SIRT6 activity.^9^ Conversely, recent studies also described SIRT6 as a tumor promoter, hence highlighting the context-dependent role of this enzyme in cellular homeostasis.^10,11^

Homozygous mutations leading to SIRT6 loss of activity in humans caused fetal loss associated with muscle and brain developmental deficiencies.^12^ Similarly, cynomolgus monkeys bearing a SIRT6 knockout obtained through CRISPR-Cas9 suggested a primary role of SIRT6 for primates’ fetal development.^13^

Conversely, SIRT6 overexpression in male mice determined an increased lifespan, and another study indicated that SIRT6 levels increase in cultured cells, mice, and rats under conditions of caloric restriction, a dietary program that protects against many aging-related changes.^14^

SIRT6 has been initially described as an HDAC, having histone H3 as a substrate and catalyzing the deacetylation of lysines Lys9, Lys18, and Lys56.^15−18^ Histone deacetylation is associated with compaction of chromatin and consequent transcriptional repression as well as DNA-damage response. Nevertheless, recent reports indicated that SIRT6 deacetylase catalytic activity is 100 to 1000 times lower compared to the most active SIRTs.^19^ In addition, the deacylase efficiency of SIRT6 has been shown to be higher compared to deacetylation, which can be in turn activated by small molecules, including free fatty acids (FFAs).^20,21^ For instance, *in vitro* demyristoylation activity is roughly 300 times higher than deacetylation.^22^ Nonetheless, the majority of studies on SIRT6 indicate deacetylation as the main reaction responsible for its cellular functions, while deacylation has only been reported in the case of TNF-α^22^ and R-Ras2^23^ so far. These features, along with the ability of SIRT6 to catalyze mono-ADP-ribosylation, depict a complicated picture of SIRT6 biological functions. Many details connecting the biochemical activity of SIRT6 and the observed phenotypes in both physiological and pathological conditions are still missing; hence, the main goals for future investigations consist of uncovering new SIRT6 substrates and elucidating its molecular interactors.

An important strategy for further elucidation of SIRT6 activity is played by chemical probes that through activation or inhibition of SIRT6 enzymatic activity may help to clarify the connection between SIRT6 function and the observed phenotypes. In addition, given the central role that SIRT6 plays in processes such as DNA repair, metabolism, aging, and tumorigenesis, small-molecule modulators could represent potential weapons for SIRT6-targeted treatment of diseases such as diabetes, obesity, cancer, and neurodegeneration.

## SIRT6 Structure and Catalytic Mechanism

**Figure 2:**
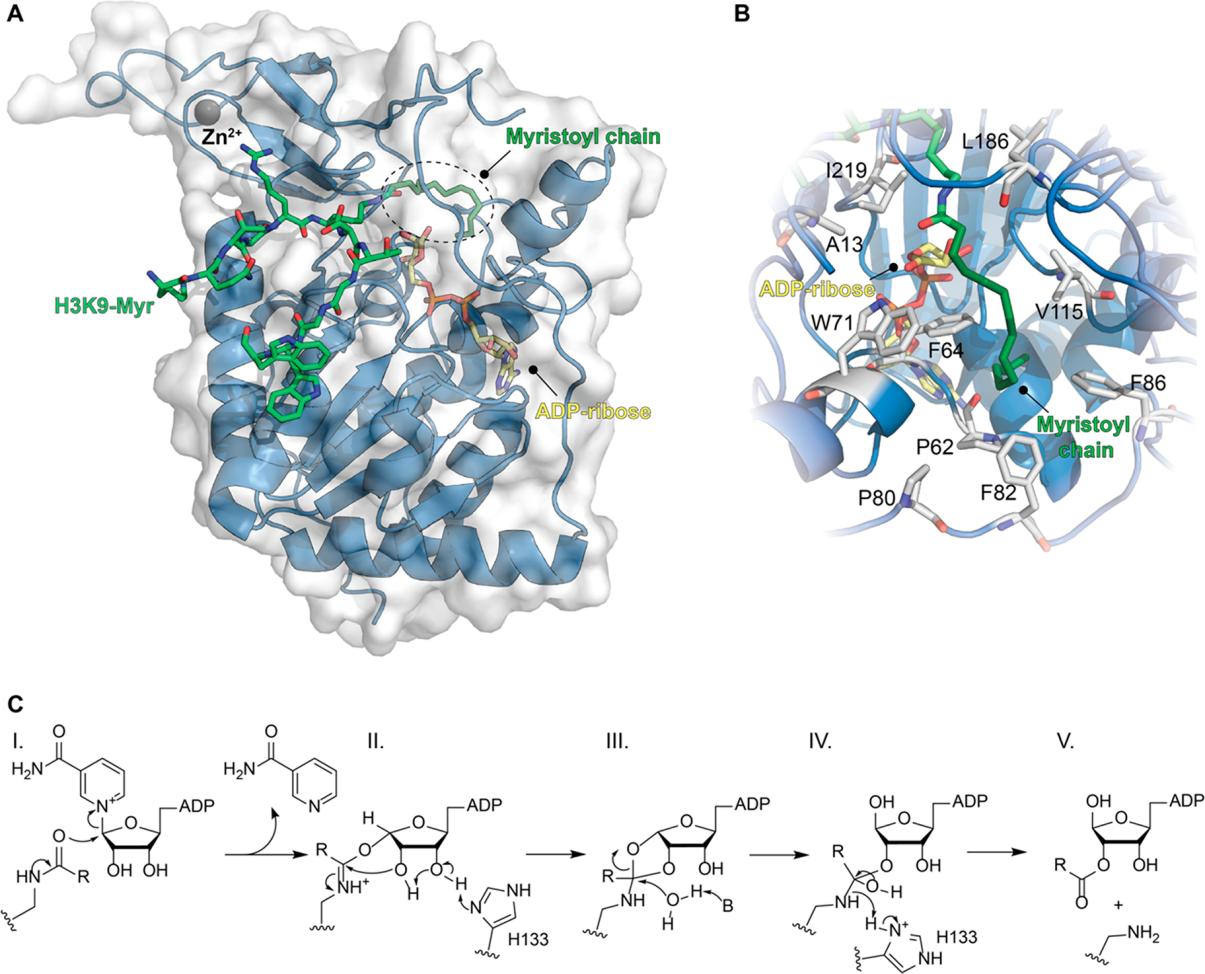
(A) Structure of SIRT6 in complex with H3K9-Myr (green) and ADP–ribose (yellow) bound (PDB ID: 3ZG6). (B) Focus on the hydrophobic pocket in the Rossman fold accommodating the myristoyl chain. (C) Catalytic mechanism of SIRT6-mediated deacylation.

A key role in the investigation of SIRT6 function is played by the elucidation of its structural features. A decade has passed since the first structure of SIRT6 in complex with ADP–ribose has been solved,^19^ followed by the structure of SIRT6 bound to both ADP–ribose and myristoylated H3K9 peptide (Figure 2A).^22^ SIRT6 has two globular domains: a large Rossmann fold and a small zinc-binding region. The Rossman fold consists of six β-sheets sandwiched between four α-helices on one side and two α-helices on the other side. This domain contains the NAD^+^ binding site as well as a hydrophobic pocket to accommodate the acyl chains of SIRT6 substrates. Differently from other SIRTs, SIRT6 has been reported to bind NAD^+^ in the absence of the acylated substrate.^19^ This feature is explained by the structural differences in the NAD^+^-binding region. Indeed, SIRT6 lacks the cofactor-binding loop^24−26^ but presents a helix (α3) that keeps its ordered structure even in the absence of the acylated peptide.^19^

The hydrophobic channel is shaped by residues belonging to different loops engaging hydrophobic interactions with the fatty acyl chain, as shown by Lin and colleagues (Figure 2B).^22^ In the presence of a myristoylated peptide, the *N*-terminus of SIRT6, which covers part of the hydrophobic pocket, becomes structured. The structural ordering induced by the myristoylated peptide may facilitate the catalytic process and explain the higher catalytic efficiency of long-fatty-chain deacylation compared to deacetylation.^22^ It can also account for the increased deacetylation activity in the presence of FFA and small molecules. Nonetheless, there are no SIRT6 structures bound to an acetylated substrate; hence, this hypothesis is yet to be proven.

Finally, the zinc-binding motif is only structural and does not participate in the catalysis; this feature is shared by all SIRTs and differentiates them from class I, II, and IV HDACs possessing in the active site a zinc ion essential for catalysis.^27^

Notably, the *in vitro* deacetylase activity of SIRT6 is much lower than that of other SIRTs, probably because of SIRT6 peculiar structural features. Nevertheless, several cell-based assays suggested that the deacetylation is the most prominent activity of SIRT6, and H3K9 was indicated as the SIRT6 main substrate.^19^ This is explained by the fact that SIRT6 preferably associates with histones when they are packaged in nucleosomes. Conversely, SIRT1 exhibits higher deacetylation activity toward unpacked histones. Thus, interaction with packaged histones may trigger a transition toward an active SIRT6 conformation. Hence, SIRT6 activity depends on histone packaging, thereby being lower when tested *in vitro* using free histones.^28^ It is therefore possible that in the case of other substrates SIRT6 deacetylase activity is affected by the presence of interactors contributing to the formation of multiprotein complexes.

As mentioned above, SIRT6 deacylase activity has been reported to be higher than deacetylation.^22^ However, a functional role for SIRT6-mediated deacylation has only been described in the case of TNF-α^22^ and R-Ras2.^23^ Importantly, histone deacylation has only been indicated in preliminary *in vitro* studies. In the same study in which TNF-α deacylation was described for the first time, Jiang et al. also showed that SIRT6 can catalyze the removal of octanoyl, myristoyl, and palmitoyl groups from H3K9 and of myristoyl from H2BK12 using synthetic histone peptides as substrates.^22^ A subsequent analysis was performed using a chemical biology approach, in which the SIRT6-acylated substrate was the octenoylated H3 incorporated in the nucleosome. The terminal olefin selectively could react with a tetrazine probe allowing nucleosome labeling. This study suggested that SIRT6 catalyzes the efficient deacylation of H3K9, H3K18, and H3K27 while having low activity toward H3K4 and H3K23.^29^ Nonetheless, the precise physiological role of histones’ acylation/deacylation equilibria need further elucidation.

The SIRT6 residue Gly60 is pivotal for deacetylation; indeed, the G60A mutant has its deacetylase activity abolished while retaining deacylase activity. Mechanistically, Gly60 is crucial for NAD^+^ binding, and fatty-acylated substrates, but not acetylated ones, are able to reverse the conformational change induced by its mutation.^30^ This is in line with the above-mentioned activation of SIRT6-mediated deacetylation in the presence of FFA.^20,21^

Beyond deacetylation and deacylation, SIRT6 also catalyzes mono-ADP-ribosylation. This was initially demonstrated using mouse SIRT6 (mSIRT6), which was shown to self-mono-ADP-ribosylate, and suggested that SIRT6 may self-modulate its activity through this post-translational modification (PTM).^4^ Further studies indicated that SIRT6 mono-ADP-ribosylates different factors, including the poly(ADP–ribose) polymerase 1 (PARP1),^31^ the transcription factor KAP1,^32^ the BAF chromatin remodeling complex subunit BAF170,^33^ and the histone lysine demethylase KDM2A.^34^

Given its peculiar structure, SIRT6 can bind NAD^+^ before the acylated protein, and following binding of both substrates, a slow conformational change allows the formation of the alkylimidate intermediate (Figure 2C, step I). The rate of this step is enhanced by FFA and small molecules and has been shown to be faster during demyristoylation.^21^ This reaction consists of a nucleophilic attack of the acyl carbonyl on C1′ of nicotinamide-bound ribose and consequent formation of a C1′-*O*-alkylimidate intermediate, along with release of nicotinamide. Subsequently, His133 acts as a general base on ribose C3′ thereby triggering an intramolecular nucleophilic attack of the C2′ hydroxyl toward the C1′-*O*-alkylimidate, thus yielding the C1′-C2′ cyclic intermediate (Figure 2C, step II). A conserved water molecule then catalyzes the hydrolysis of the cyclic intermediate, affording the tetrahedral intermediate (Figure 2C, step III). The imino group then attacks His133, which is now positively charged, thus gaining a proton and resulting in the cleavage of the C–N bond. This leads to the final products *O*-acyl-ADP–ribose and deacylated lysine (Figure 2C, step IV), which are then released from the enzyme (Figure 2C, step V).^3,21^

## Biological Functions and Disease Relevance of SIRT6

### Genome Maintenance

Initial observations on SIRT6-knockout mice revealed hypersensitivity to DNA-damaging agents and genomic instability, indicating aberrant functioning of DNA double-strand break (DSB) repair and base excision repair (BER) mechanisms.^35^ Following these early studies, a growing body of reports indicated that SIRT6 associates with damaged chromatin sites^36^ and coordinates the recruitment of different factors to initiate DNA-damage repair (DDR).^36−39^ In particular, Onn and colleagues suggested that a SIRT6 dimer is able to directly bind to open-ended DSBs, whereby each monomer interacts with one DNA strand.^40^

SIRT6-mediated recruitment of repair factors is triggered by the deacetylation of nucleosomes. For instance, H3K56 deacetylation facilitates the recruitment of the ISWI-chromatin remodeller SNF2H, which increases chromatin accessibility, thereby promoting the binding to damaged DNA of repair factors such as BRCA1, RPA, and 53BP1.^37^ Remarkably, a recent study indicated the crucial role of the SIRT6-SNF2H dimer at the neurological level. Indeed, animals lacking SIRT6 in the brain showed AD symptoms, including increased levels of hyperphosphorylated Tau protein.^41^

**Figure 3:**
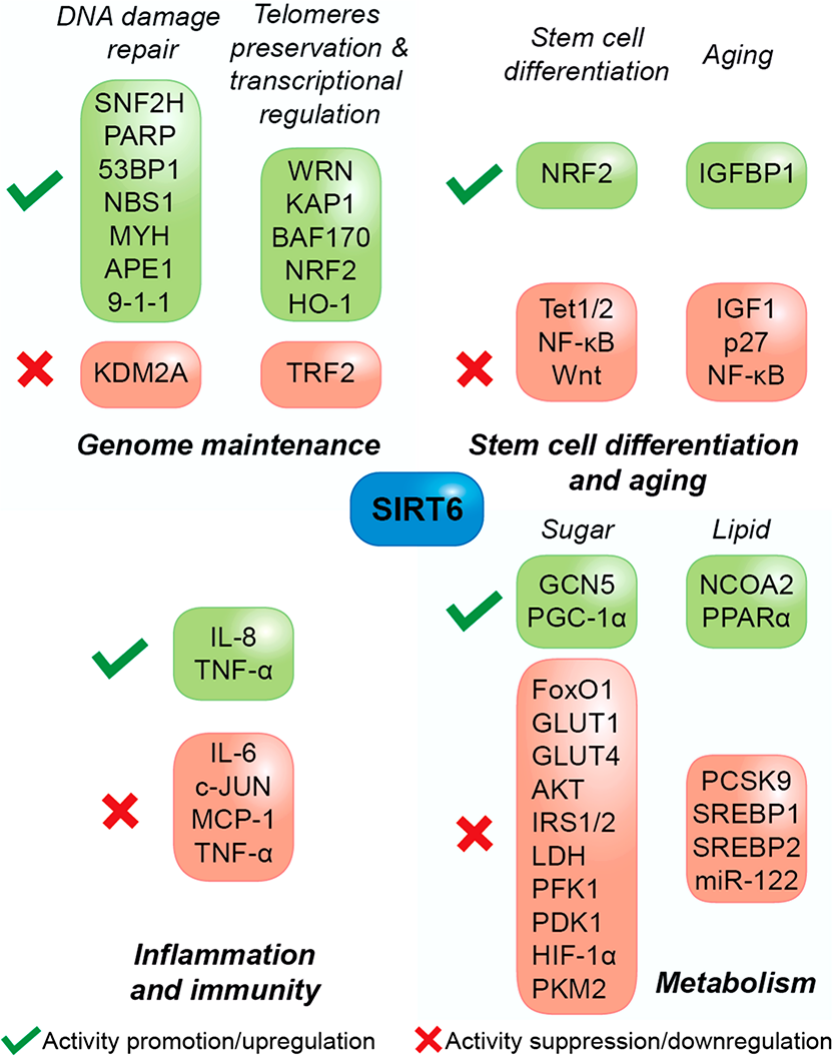
Roles of SIRT6 in cellular and organism homeostasis. The figure indicates the main proteins involved in the most important processes regulated by SIRT6, except cancer.

Recent studies indicated that SIRT6 is phosphorylated on Ser10 by *c*-Jun *N*-terminal kinase (JNK) under oxidative stress conditions. This PTM enables the binding of SIRT6 to DSBs and subsequent recruitment of PARP1, which mediates nonhomologous end-joining (NHEJ) and homology-directed repair (HDR). PARP1 is also mono-ADP-ribosylated by SIRT6 on Lys521,^31^ a modification that is required for PARP1 activity in BER. In addition, mono-ADP ribosylation of the histone lysine demethylase KDM2A has been shown to augment H3K36me2 level at DNA-damage sites, thereby promoting H3K9 trimethylation and consequent recruitment of NHEJ factors to DSBs (Figure 3).^34^

As anticipated above, the role of SIRT6 in DNA repair has implications in pathology and therapy, particularly in neurodegeneration as the frequency and precision of repair mechanisms declines with age. Accordingly, SIRT6 levels have been shown to decrease with cellular senescence and its overexpression is able to stimulate HDR through the PARP1 pathway.

SIRT6 mediates DNA repair also through BER as indicated by reports showing that overexpression of SIRT6 increases 2-fold the efficiency of this DNA repair mechanism.^42^ Moreover, under oxidative DNA damage, SIRT6 interacts with and stimulates MYH DNA glycosylase and the endonuclease APE1, two enzymes involved in BER. The process is aided by the checkpoint clamp Rad9-Rad1-Hus1 (9-1-1), which forms a multiprotein complex with MYH, APE1, and SIRT6 that is pivotal for whole genome and telomere stability in mammalian cells (Figure 3).^43^

SIRT6 is also responsible for telomeric preservation in mammalian cells through deacetylation of H3K9 and H3K56 in telomeric regions.^15,16^ SIRT6-mediated H3K9 deacetylation determines chromatin conformational changes that allow the binding of the Werner syndrome ATP-dependent helicase (WRN), the DNA-processing factor that is mutated in the Werner syndrome, a premature aging disorder.^15^ Furthermore, SIRT6 interacts with telomere repeat binding factor 2 (TRF2), a pivotal regulator of telomere homeostasis and DNA-damage response, and their interaction is increased during DNA-damage events, in a PARP1-dependent manner. SIRT6 catalyzes TRF2 deacetylation, triggering its ubiquitination finally leading to its proteolysis. In line with this, the levels of the two proteins were negatively correlated in a cohort of colorectal cancer (CRC) patients. These results indicate a regulation mechanism of TRF2 levels in response to DNA damage and oncogenesis, whereby SIRT6-induced degradation of TRF2 impairs DNA-damage repair leading to cancer cell death.^44^

Apart from its roles in DNA repair and telomeres, SIRT6 is mainly responsible for transcriptional silencing. SIRT6-mediated H3K9 and H3K56 deacetylation contributes to the repression of proteins involved in lipid metabolism, inflammation (NF-κB-dependent proteins), as well as c-Myc targets, ribosomal proteins, and early developmental genes.^8,45−47^ Furthermore, SIRT6 promotes the silencing of long interspersed element-1 (LINE-1) retrotransposable elements (RTEs), a class of retrotransposons linked to mutagenesis and genomic instability.^48^ SIRT6 facilitates heterochromatin packaging of these RTEs, hence suppressing transposition. Notably, recent findings indicate that this function is directed by SIRT6-mediated mono-ADP-ribosylation of the transcriptional corepressor KAP1 (Figure 3).^32^

Moreover, SIRT6 is responsible for pericentric chromatin silencing through H3K18 deacetylation, and this function is mediated by KAP1, although in a different manner compared to LINE-1 RTEs. Evidence indicates that H3K18 deacetylation is necessary for KAP1 retention at pericentric satellite repeats and consequent transcriptional repression. Conversely, SIRT6 knockout and consequent H3K18 hyperacetylation causes KAP1 detachment and transcriptional derepression.^18^ SIRT6-deficient cells display accumulation of pathological pericentric transcripts causing genomic instability, mitotic errors, and cellular senescence, defects associated with aging and tumorigenesis.

As previously mentioned, SIRT6 has also been indicated to catalyze the ADP-ribosylation of the BAF chromatin remodeling complex subunit BAF170 at Lys312. This modification enhances the transcription upon oxidative stress of a subset of the nuclear factor erythroid 2-related factor (NRF2) responsive genes such as HO-1.^33^

### Stem Cell Differentiation

Embryonic stem cell (ESC) pluripotency maintenance is guaranteed by the expression of Oct4, Sox2, and Nanog genes, which are lost upon differentiation.^49,50^ Recent studies indicated that SIRT6 mediates ESC differentiation through H3K9 and H3K56 deacetylation, which determines the repression of ten-eleven translocation methylcytosine dioxygenase 1 and 2 (TET1 and TET2). These enzymes convert 5-methylcytosine (5-mC) into 5-hydroxymethylcytosine (5-hmC) and regulate cell lineage choice during ESC differentiation (Figure 3).^51^

SIRT6 has also a role in mesenchymal stem cell (MSC) and hematopoietic stem cell (HSC) homeostasis through H3K56 deacetylation. Through this activity, SIRT6 seems to coactivate transcription of NRF2 target genes and protect MSCs from oxidative stress.^52^ In addition, SIRT6-mediated H3K56 deacetylation was shown to suppress the NF-κB signaling pathway, thereby promoting osteogenic differentiation and new bone formation and repair in rats.^53^ In case of HSCs, SIRT6 interacts with LEF1 and, through H3K56 deacylation, corepresses Wnt target genes, thus blocking aberrant HSC proliferation.^54^

In addition, SIRT6 expression is associated with higher reprogramming efficiency of induced pluripotent stem cells (iPSCs).^55^ Given the increasing evidence supporting iPSC-based therapies in the context of neurodegenerative diseases, SIRT6 activation may represent a useful strategy to increase the success rate of these treatments.

### Aging

Given its roles in genomic maintenance and stem cell homeostasis, SIRT6 has an indirect influence on aging, a process tightly related to DNA damage, telomere maintenance, and differentiation. In addition, SIRT6 plays a direct role in senescence and aging-related conditions through its activity on specific substrates at both the cytoplasmic and nuclear level.

SIRT6 overexpression determined a 15% increase of male mice life expectancy along with reduction of insulin-like growth factor 1 (IGF1) signaling through increased levels of IGF-binding protein 1 (IGFBP1) and altered phosphorylation of proteins involved in IGF1 downstream signaling.^7^ Moreover, SIRT6 deacetylates the cyclin-dependent kinase inhibitor p27, a factor involved in cellular senescence, hence promoting its proteasome-dependent degradation.^56^ Similarly, the transcription factor NF-κB, which induces the expression of aging-related genes, is negatively regulated by SIRT6 through a double mechanism. Indeed, at the transcriptional level, SIRT6 deacetylates H3K9 at NF-κB promoters, thereby reducing the expression of its components, while at the protein level, SIRT6 catalyzed deacetylation of the NF-κB p65 subunit (RelA) at Lys310 results in NF-κB nuclear export (Figure 3).^57^

### Cancer

**Figure 4:**
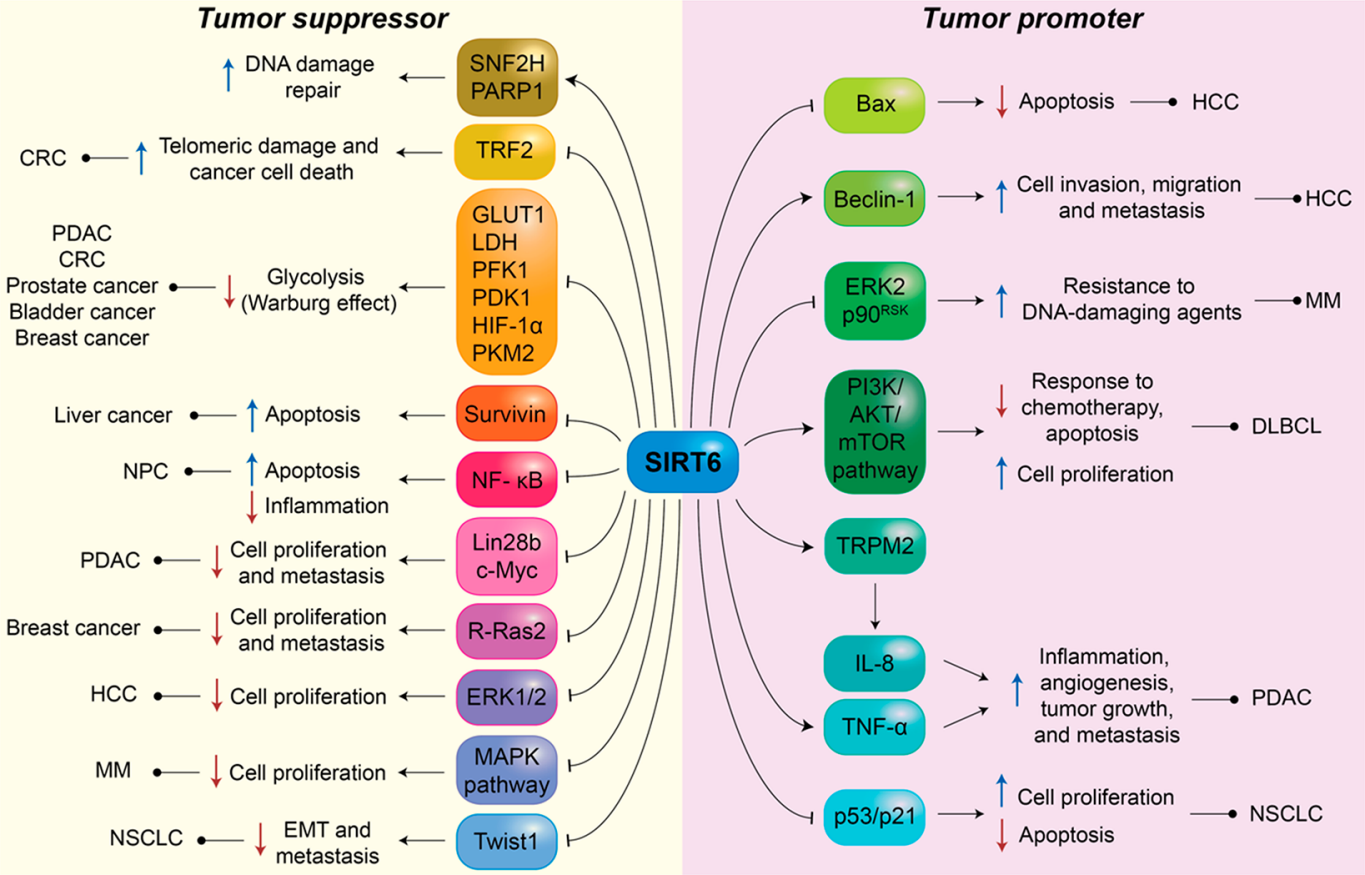
Roles of SIRT6 in cancer. The figure indicates the main factors modulated by SIRT6 in the context of both tumor suppression and promotion. Different mechanisms are involved, including the regulation of DNA-damage response, glycolysis, apoptosis, cell migration, and inflammation.

DNA damage and cell cycle dysregulation are two of the most important hallmarks of cancer; hence, it comes with no surprise that SIRT6 has been regarded as a tumor suppressor, as indicated by early studies in knockout mice showing genomic instability.^6^ Further investigations indicated that SIRT6 knockout in MEFs leads to tumorigenesis without activation of known oncogenes, and deletion of SIRT6 *in vivo* correlates with an increased number, size, and aggressiveness of tumors (Figure 4).^8,11^ The tumor-suppressor role of SIRT6 was associated with the suppression of glycolytic genes crucial for the Warburg effect, a metabolic shift common in cancer cells where ATP is obtained mostly through glycolysis rather than mitochondrial oxidative phosphorylation, in order to generate immediate energy to support fast proliferation and related cellular processes.^58^ These genes, including the glucose transporter-1 (GLUT1), lactate dehydrogenase (LDH), phosphofructokinase-1 (PFK1), and pyruvate dehydrogenase kinase-1 (PDK1), are regulated by the hypoxia-inducible factor 1α (HIF-1α), which is corepressed by SIRT6.^59^ SIRT6 also deacetylates pyruvate kinase M2 (PKM2), a nuclear isozyme that enhances aerobic glycolysis even under hypoxia conditions and promotes tumor growth. SIRT6-mediated deacetylation triggers PKM2 transport to the cytoplasm and repression of its functions.^60^ In addition, glycolytic genes are downregulated through direct deacetylation of H3K9 at their promoters.^59^

In line with this, SIRT6 is selectively downregulated in CRC and pancreatic ductal adenocarcinoma (PDAC), which display increased expression of glycolysis-related genes.^8^ The following studies confirmed these findings and expanded the role of SIRT6 as a main regulator of glycolysis in prostate, bladder,^61^ and breast cancers.^62^ Interestingly, SIRT6 activity is antagonized by the Runt-related transcription factor 2 (RUNX2), which represses SIRT6 transcription in low-glucose conditions.^62^ In addition, E2 transcription factor 1 (E2F1) negatively regulates SIRT6 in response to hypoxia, hence facilitating the Warburg effect.^61^

SIRT6-mediated H3K9 deacetylation has effects on multiple oncogenes beyond glycolytic genes. These include two proteins involved in apoptosis inhibition and consequently tumor progression: the caspase activation inhibitor survivin^63^ and the RNA-binding oncofetal protein Lin28b.^64^ Liver cancer mouse models also showed that survivin activity is impaired through the inhibition of NF-κB activation and consequent binding to a survivin promoter.^65^ NF-κB is also involved in the activation of other antiapoptotic proteins (FLIP, c-IAP1/2, and XIAP)^66^ and its expression is antagonized by SIRT6 in nasopharyngeal carcinoma (NPC).^67^ Lin28b expression is downregulated through deacetylation of both H3K9 and H3K56. In PDAC, SIRT6 deficiency was associated with H3K9 and H3K56 hyperacetylation at the Lin28b promoter and poor patient prognosis; moreover, in a mouse model of pancreatic cancer, a SIRT6 deficit led to increased tumor aggressiveness and metastasis.^68^ Given the severity of PDAC and the important role played by SIRT6 in this subset of tumors, targeting this pathway through activation of SIRT6 may represent a successful approach for this type of malignancy.

Lin28b is also a target gene of the c-Myc oncogene. In PDAC, Lin28b promoter hyperacetylation is associated with c-Myc recruitment and consequent augmentation of cancer progression and metastasis.^68^ Notably, c-Myc activity is antagonized by SIRT6, which represses the transcription of c-Myc and its target genes and leads to cell cycle arrest and inhibition of tumor growth.^69,70^

SIRT6 deacylase activity also contributes to its action as a tumor suppressor. Indeed, SIRT6 deacylates the GTPase R-Ras2, a Ras-family protein that contributes to tumorigenesis and metastasis.^23^ SIRT6-mediated deacylation of R-Ras2 shifts its location toward intracellular vesicles rather than the plasma membrane, where it usually sits, hence blocking its signaling and cell proliferation.^23^

Notwithstanding the great number of reports indicating the tumor-suppressor role of SIRT6 in many forms of cancer, some evidence points toward an oncogenic role of SIRT6 under specific conditions (Figure 4). For instance, in hepatocellular carcinoma (HCC), the activity of SIRT6 in DNA-damage repair and cellular senescence prevention play in favor of cancer cell growth.^71,72^ In addition, SIRT6 deacetylates H3K9 at the promoter of the proapoptotic factor Bax, resulting in evasion from apoptosis.^73^ SIRT6 also facilitates the epithelial–mesenchymal transition (EMT) in HCC through deacetylation of the autophagy regulator Beclin-1 leading to the autophagic degradation of E-cadherin, a crucial receptor involved in cell adhesion.^74^ The controversial role of SIRT6 in cancer is confirmed by a separate study that suggests a protective role of SIRT6 in HCC mediated by the inhibition of ERK1/2 phosphorylation and the suppression of its downstream pathway.^75^

A connection between SIRT6 and MAPK signaling has also been described in multiple myeloma (MM), although at a transcriptional level. In MM, high SIRT6 levels have been associated with poor prognosis. This disease is characterized by high genome instability; hence, SIRT6 overexpression may be a compensatory response to facilitate DNA repair. While this mechanism may be favorable for cancer cell survival, Cea and colleagues demonstrated that in MM cells, SIRT6 interacts with ELK1 and deacetylates H3K9 at the promoters of MAPK signaling genes, thus stopping cell proliferation. On the other hand, SIRT6-mediated suppression of ERK2 and p90^RSK^ signaling increases resistance to DNA-damaging therapeutics.^76^ Upregulation of SIRT6 has also been observed in other blood cancers, including acute myeloid leukemia,^77^ chronic lymphocytic leukemia,^78^ and diffuse large B-cell lymphoma (DLBCL).^79^ In DLBCL cells, knockdown of SIRT6 leads to higher sensitivity to chemotherapy, altered cell proliferation, augmented rates of apoptosis, and cell cycle arrest. These phenotypes were associated with inhibition of the PI3K/AKT/mTOR signaling pathway.^79^

SIRT6 catalytic activity determines an increase of the intracellular ADP–ribose concentration, which activates the Ca^2+^ channel TRPM2. Increased Ca^2+^ concentration finally leads to the activation of the Ca^2+^-dependent nuclear factor of activated T cells (NFAT), which upregulates the expression of TNF-α and IL-8, two proangiogenetic and proinflammatory cytokines that promote tumor growth and metastasis.^80^

A recent study indicated that SIRT6 is overexpressed in NSCLC cells, and its silencing determined activation of the p53/p21 pathway and consequent inhibition of cell proliferation associated with cell cycle arrest and apoptosis.^81^ Conversely, an earlier study indicated that SIRT6 suppresses NSCLC proliferation through inhibition of Twist1 expression, a factor that facilitates EMT and metastasis.^82^

These examples indicate the complicated role played by SIRT6 in tumorigenesis, suggesting a context dependency. If we take into account the involvement of SIRT6 in DNA-damage repair, depending on the stage of cancer progression, this pathway may have tumor-promoting or tumor-suppressing effects.^11^ Moreover, high levels of SIRT6 associated with tumors may also represent a compensating response rather than a causality. Therefore, it is vital to distinguish the potential of SIRT6 as a therapeutic target or as a biomarker in each type of tumor.

### Inflammation and Immunity

In the context of immune regulation, SIRT6 has been shown to upregulate proinflammatory cytokines, as explained above in the case of TNF-α and IL-8.^80^ SIRT6 catalyzes the demyristoylation of TNF-α at Lys19 and Lys20, triggering its secretion^22,30^ during inflammatory response, while acylated TNF-α is retained and finally degraded in lysosomes. In addition, TNF-α levels are positively regulated by NAD^+^ concentration, and SIRT6 was identified as the mediator of the increased translation efficiency of *Tnf* mRNA.^83^

On the other hand, SIRT6 also exerts anti-inflammatory roles through negative regulation of NF-κB, and this is supported by studies in macrophages where SIRT6 deletion promotes NF-κB activation and IL-6 production.^84^ Studies performed on SIRT6-knockout mice indicated a chronic liver inflammation and fibrosis. Moreover, SIRT6-deficient lymphocytes and myeloid-derived cells presented aberrant activation. Mechanistically, SIRT6 repressed the transcription of genes controlled by the oncogenic transcription factor c-JUN (Figure 3).^85^

### Sugar and Lipid Metabolism

SIRT6 is undoubtedly a multitasking protein, and beyond its involvement in DNA maintenance and cancer progression, its main function is probably the regulation of glucose and lipid metabolism. As outlined in the context of cancer, SIRT6 corepresses HIF-1α and deacetylates H3K9 at glycolytic genes promoters,^59^ thus channeling glucose catabolism from glycolysis toward more energy-efficient pathways (Figure 4). Indeed, SIRT6-knockout mice display increased glycolytic pathway associated with high glucose uptake, increased insulin signaling, and severe hypoglycemia as a compensatory response.^6,59^

SIRT6 modulates glucose homeostasis also through control of gluconeogenesis. SIRT6 has been found to deacetylate the acetyltransferase general control nonderepressible 5 (GCN5), a regulator of cell cycle progression involved in the onset of different tumors,^86−88^ leading to an increased enzymatic activity. In the liver, increased GCN5 activity results in acetylated PGC-1α,^89^ thus leading to reduced gluconeogenesis gene expression,^90^ which prevents hyperglycemia in diabetic/obese mice.^89^ Another important transcription factor for gluconeogenesis is FoxO1, which activates the transcription of the rate-limiting gluconeogenesis enzymes glucose-6-phosphatase (G6PC) and phosphoenolpyruvate carboxykinase (PCK1).^91^ FoxO1 is deacetylated by SIRT6, triggering its nuclear export and reduced transcription of its target genes (Figure 3).^92^

SIRT6 activity has also an effect on insulin signaling through downregulation of glucose transporters GLUT1 and GLUT4 and decreased phosphorylation of AKT,^93^ an important regulator of cellular glucose uptake.^94^ Mechanistically, SIRT6 is involved in the inactivation of AKT upstream proteins, including the insulin receptor substrates IRS1/2.^93^ Therefore, the absence of SIRT6 sensitizes the organism to insulin action, giving a complementary explanation to glycolytic gene suppression for the observed hypoglycemia in SIRT6-knockout mice.

In the case of lipid metabolism, SIRT6 reduces triglyceride synthesis and fatty-acid uptake while promoting β-oxidation, as indicated by mice-knockout studies.^47^ SIRT6 also contributes to keeping low the levels of LDL-C (Figure 3).^95^ Mechanistically, SIRT6 has been shown to decrease acetylation of the PPARα coactivator NCOA2, although it is not clear whether NCOA2 is a direct substrate of SIRT6 enzymatic activity. This determines activation of PPARα, a key transcription factor for hepatic β-oxidation genes.^96^ Furthermore, SIRT6 represses the expression of the proprotein convertase subtilisin/kexin type 9 (PCSK9), which controls the degradation of LDL-C in lysosomes (Figure 3).^97^ Through interaction with FoxO3α, SIRT6 is recruited at the PCSK9 promoter, where it deacetylates H3K9 and H3K56, hence suppressing its transcription.^98^ SIRT6 also deacetylates H3K9 and H3K56 at the promoters of genes regulated by the sterol regulatory element-binding protein 1 and 2 (SREBP1/2).^99^ These transcriptional regulators activate transcription of lipogenic genes and are also directly controlled by SIRT6 at the transcriptional level through H3K56 deacetylation at promoters. Notably, micro-RNAs miR-33a and mi33b, which are expressed from the introns of SREBP1/2, are associated with repression of SIRT6 levels, contributing to a negative feedback loop in the SIRT6-SREBP1/2 axis.^100^ Another micro-RNA involved in SIRT6-mediated pathways is miR-122, the most abundant hepatic miRNA, which negatively regulates SIRT6 expression and is in turn negatively regulated by SIRT6 (Figure 3). In addition, while SIRT6 positively regulates genes involved in fatty-acid β-oxidation, miR-122 performs an opposite action.^101^

The connection between SIRT6 activity and fatty-acid metabolism is fascinating given the evidence indicating that FFA are capable of increasing SIRT6 activity *in vitro*.^20^ Therefore, SIRT6 may act as a fatty-acid sensor that amplifies metabolic signals into epigenetic responses that affect crucial homeostatic mechanisms beyond metabolism itself; these include all the pathways analyzed in this section such as genomic maintenance, immunity, cellular differentiation, and transformation.

## Pharmacological Modulation of SIRT6

The implications of SIRT6 as a positive regulator of metabolism and aging, along with the discovery that the deacetylase activity may be enhanced by FFA, has stimulated research groups toward the development of SIRT6 activators (Table 1). On the other hand, given the dual role of SIRT6 in inflammation and cancer, inhibitors have also been developed (Table 2). The possibility of either activating or inhibiting SIRT6 in a context-dependent manner paves the way for personalized pharmacology. From a wider perspective, highly potent and selective SIRT6 modulators (both activators and inhibitors) allow the molecular details of its activity to be better scrutinized and further validate the enzyme as a pharmacological target.

**Table 1:**
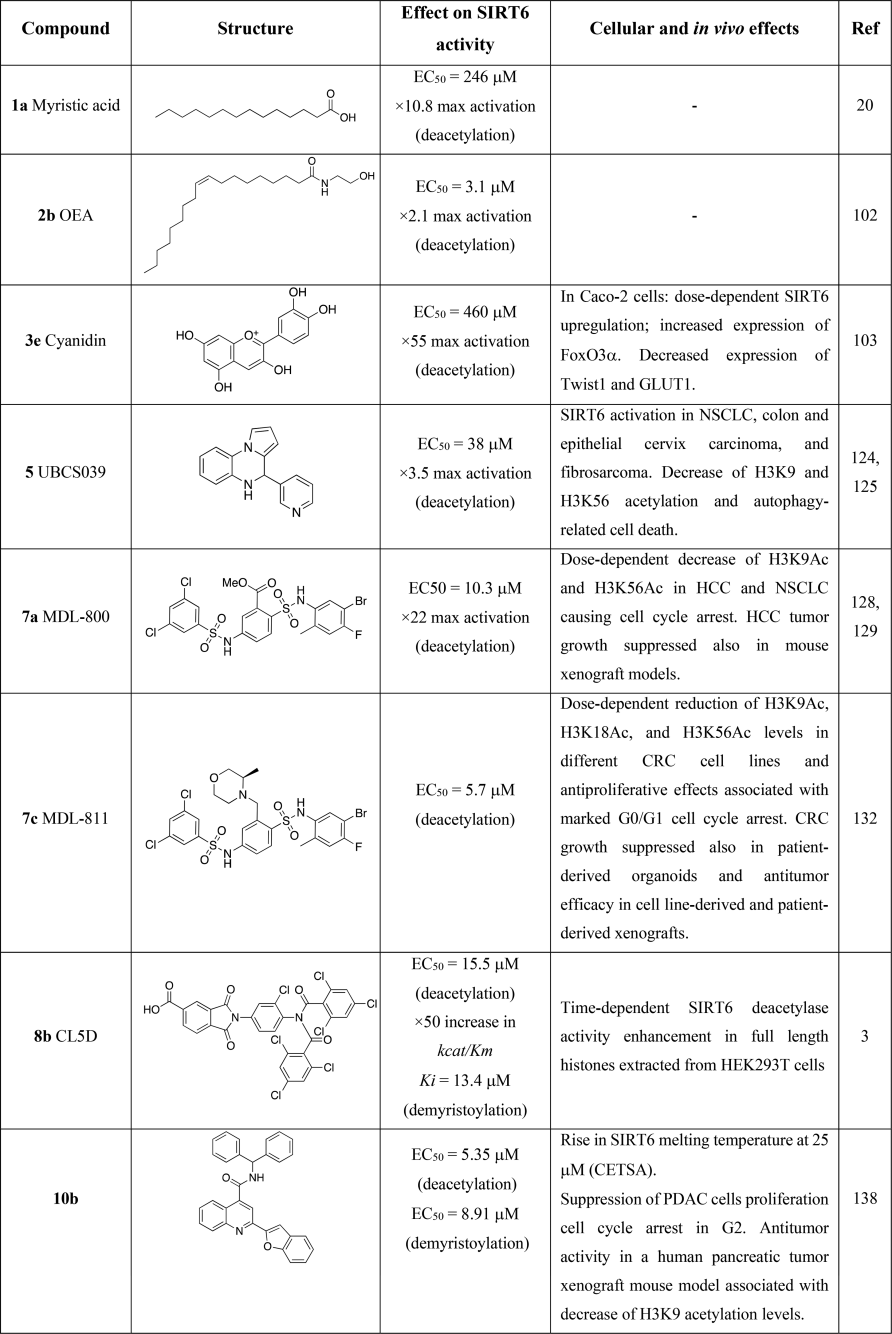
Most Relevant SIRT6 Activators

**Table 2:**
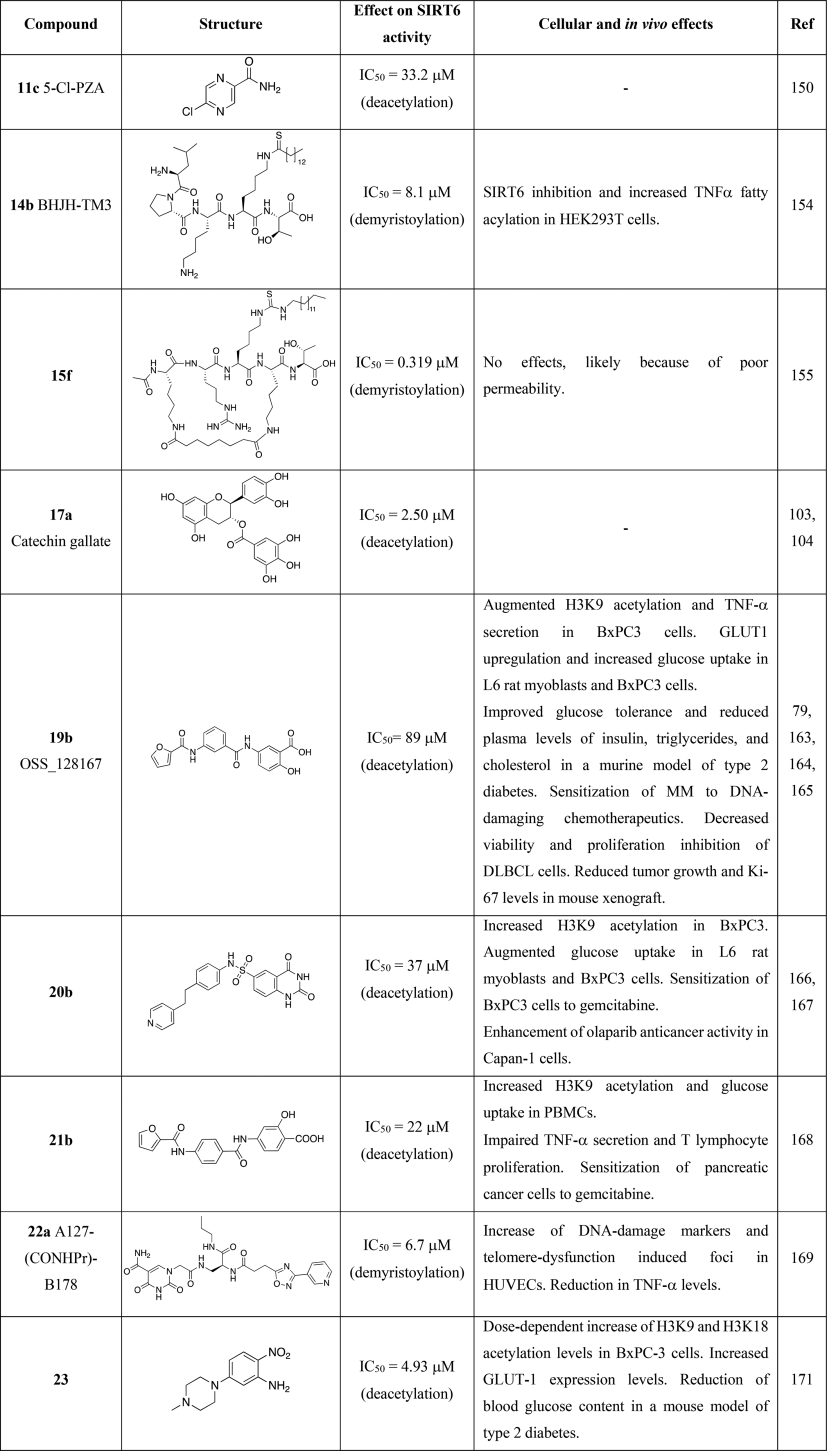
Most Relevant SIRT6 Inhibitors

In the following section, we will first discuss the most relevant SIRT6 activators followed by a detailed description of SIRT6 inhibitors.

### SIRT6 Activators

**Figure 5:**
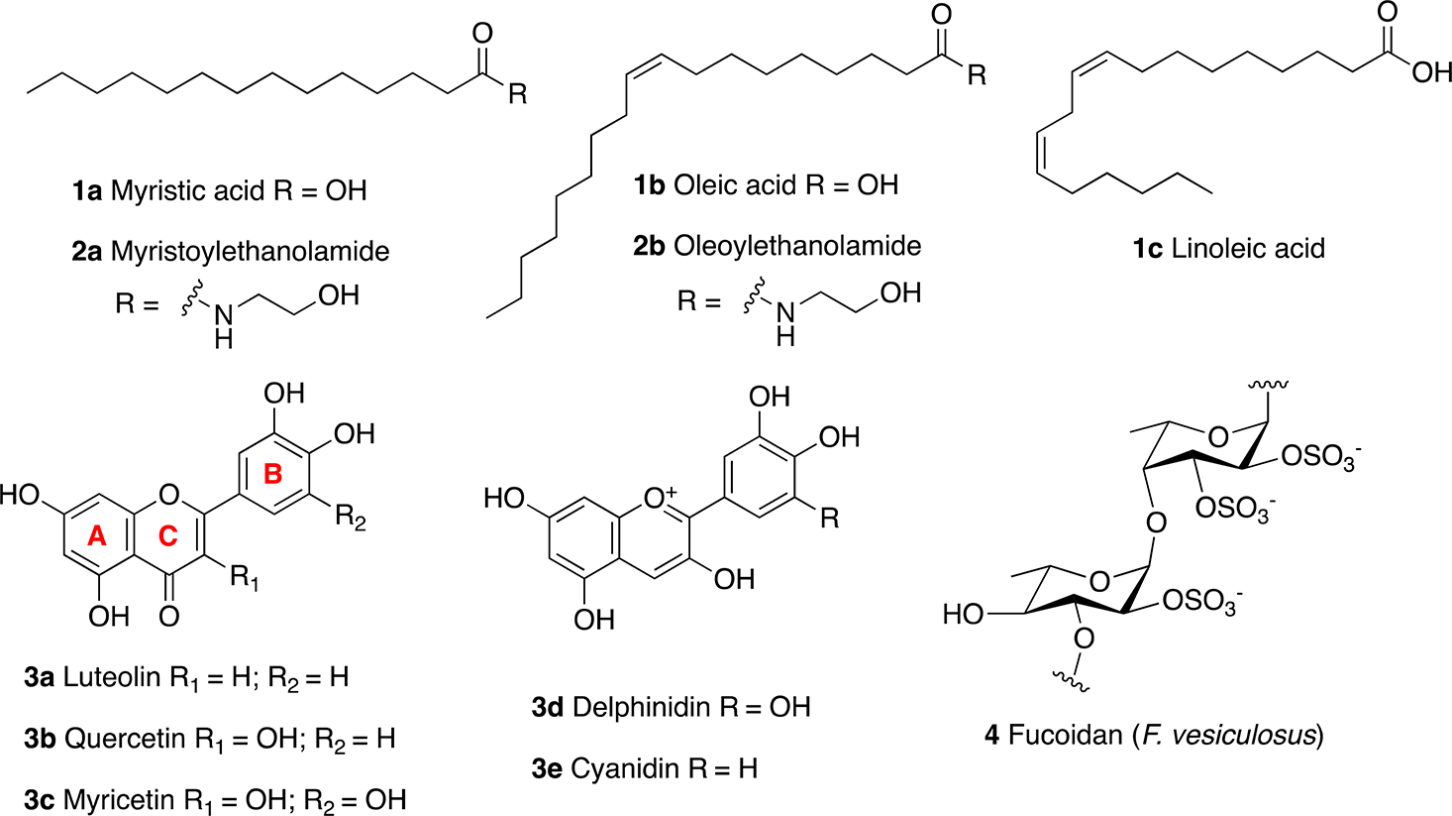
SIRT6 modulators based on endogenous ligands (upper panel) and natural products (lower panel).

As already mentioned, early studies on SIRT6 activity indicated that FFA containing 14 to 18 carbons (Figure 5, upper panel) stimulated SIRT6 activity. In particular, myristic acid (**1a**) increased deacetylase activity up to 10.8 times, with an EC_50_ of 246 μM with a 35-fold increase in catalytic efficiency (*k*_cat_/*K*_m_ value, i.e., the ability of SIRT6 to capture a substrate for catalysis) at 400 μM, suggesting increased affinity of SIRT6 for the acetylated substrate.^20^ Oleic (**1b**) and linoleic acid (**1c**) displayed EC_50_ values of 90 and 100 μM, yielding an increase in deacetylase activity of 5.8 and 6.8 times, respectively. In the same study, **1a** was shown to act as a competitive inhibitor for demyristoylation, suggesting that the same hydrophobic pocket occupied by FFA during deacetylation is necessary to accommodate the long acyl chain of fatty-acid substrates for deacylation. These findings are the basis for the development of small-molecule SIRT6 activators.^20^

Following the studies on FFA, it has been shown that myristoylethanolamide (MEA, **2a**) and oleoylethanolamide (OEA, **2b**), the ethanolamine derivatives of **1a** and **1b**, showed a 2-fold maximum activation of SIRT6 and EC_50_ values of 7.5 and 3.1 μM, respectively.^102^ In the same study, **1b** and **1c** were tested, yielding SIRT6 maximum-fold activation of 4.6 and 3.7 along with EC_50_ values of 89 and 230 μM, respectively. Rahnasto-Rilla et al. also evaluated the influence of the flavonoids luteolin (**3a**) and quercetin (**3b**) on SIRT6 activity. The skeleton of flavonoids consists of a benzene ring (A) fused with a heterocyclic pyran ring (C) presenting a further phenyl group (ring B) in position 2. All the compounds described here present hydroxyl groups on carbons 5 and 7 in ring A (Figure 5, lower panel). **3b** belongs to the subclass of flavonols and are characterized by an oxidized pyran ring, bearing a carbonyl group in position 4 and an additional hydroxyl group in position 3. Differently, **3a** is a flavon and lacks the hydroxyl group in position 3. Both compounds demonstrated a dose-dependent role, whereby they exert inhibitory activity at low concentrations with IC_50_ values of 1.9 μM (**3a**) and 24 μM (**3b**) while increasing the deacetylase activity at higher concentrations. In particular, **3a** showed a 6-fold maximum activation with an EC_50_ value of 270 μM, while **3b** yielded 10-fold maximum activation and an EC_50_ of 990 μM.^102^ Although the EC_50_ values for these two flavonoids are very high and with scarce pharmacological relevance, these results suggest multiple binding sites for small molecules, which may interact with an inhibition site at low concentrations while inducing favorable conformational changes that activate the enzyme at higher concentrations.

Following these studies, further flavonoids were tested for their capability of altering SIRT6 enzymatic activity.^103^ The flavonol myricetin (**3c**) has the same structure of **3b** with an extra hydroxyl group in 3′. This compound displayed an EC_50_ of 404 μM and 7.7-fold maximal SIRT6 activation, like observed for **3a**.

Anthocyanidins are a subgroup of flavonoids which, compared to flavonols, lack the carbonyl group in position 4 of ring B. Delphinidin (**3d**), the anthocyanidin analogue of **3c**, showed decreased activating potency, with an EC_50_ of 760 μM and 6.3-fold maximal SIRT6 activation. Remarkably, removal of the 3′ hydroxyl group in the case of cyanidin (**3e**) led to a massive increase in activation efficiency as indicated by the 55-fold maximum activation and EC_50_ of 460 μM.^103^ Notably, **3e** exhibited in-cell effects when tested on colon adenocarcinoma Caco-2 cells, with a dose-dependent SIRT6 upregulation along with modulation of SIRT6-associated genes such as FoxO3a, Twist1, and GLUT1. In particular, the authors observed a dose-dependent increase in FoxO3a expression, while Twist1 and GLUT1 were decreased.

Although the activity of these molecules toward other SIRTs was not assessed in this study, another report evaluated **3b** action on SIRT1–3, SIRT5, and SIRT6. In this case, **3b** showed a 2-fold maximum SIRT6 activation with an EC_50_ of 1.2 mM, and no inhibition was observed at low concentrations. Conversely, **3b** inhibited SIRT1–3 deacetylation activity and SIRT5-mediated desuccinylation in a concentration-dependent manner.^104^ In particular, at a 312.5 μM concentration, the enzymatic activities of SIRT1/2/3/5 were 60–70% compared to the respective controls, while SIRT6 activity was about 150%. Given their polyphenolic nature, flavonoids are known to present pleiotropic activities and have been shown to inhibit a diverse subset of enzymes. Among others, the starch digestive enzyme α-glucosidase is inhibited by compounds **3a**–**c** and **3e**(105−107) with IC_50_ values in the low–mid μM range, while α-amylase was shown to be inhibited by **3a**–**c** with IC_50_ values of ∼300 μM.^105^ Moreover, compounds **3a**–**e** have been reported to inhibit topoisomerases I and II^108−112^ and to affect the epigenetic regulation of transcription through inhibition of DNA methyltransferase 1 (DNMT1).^113,114^**3b** was also shown to suppress the activity of other epigenetic enzymes such as HDAC1^115^ and the histone acetyltransferase (HAT) p300.^116^ In contrast, a different study indicated that **3b** administration results in increased histone H3 acetylation, associated with HAT activation.^117^ Moreover, compounds **3a**–**c** have been reported to interfere with multiple bioassays, thus being classified among the pan assay interference compounds (PAINS)^118,119^ and suggesting caution in interpreting the results of biological studies on them.

**Figure 6:**
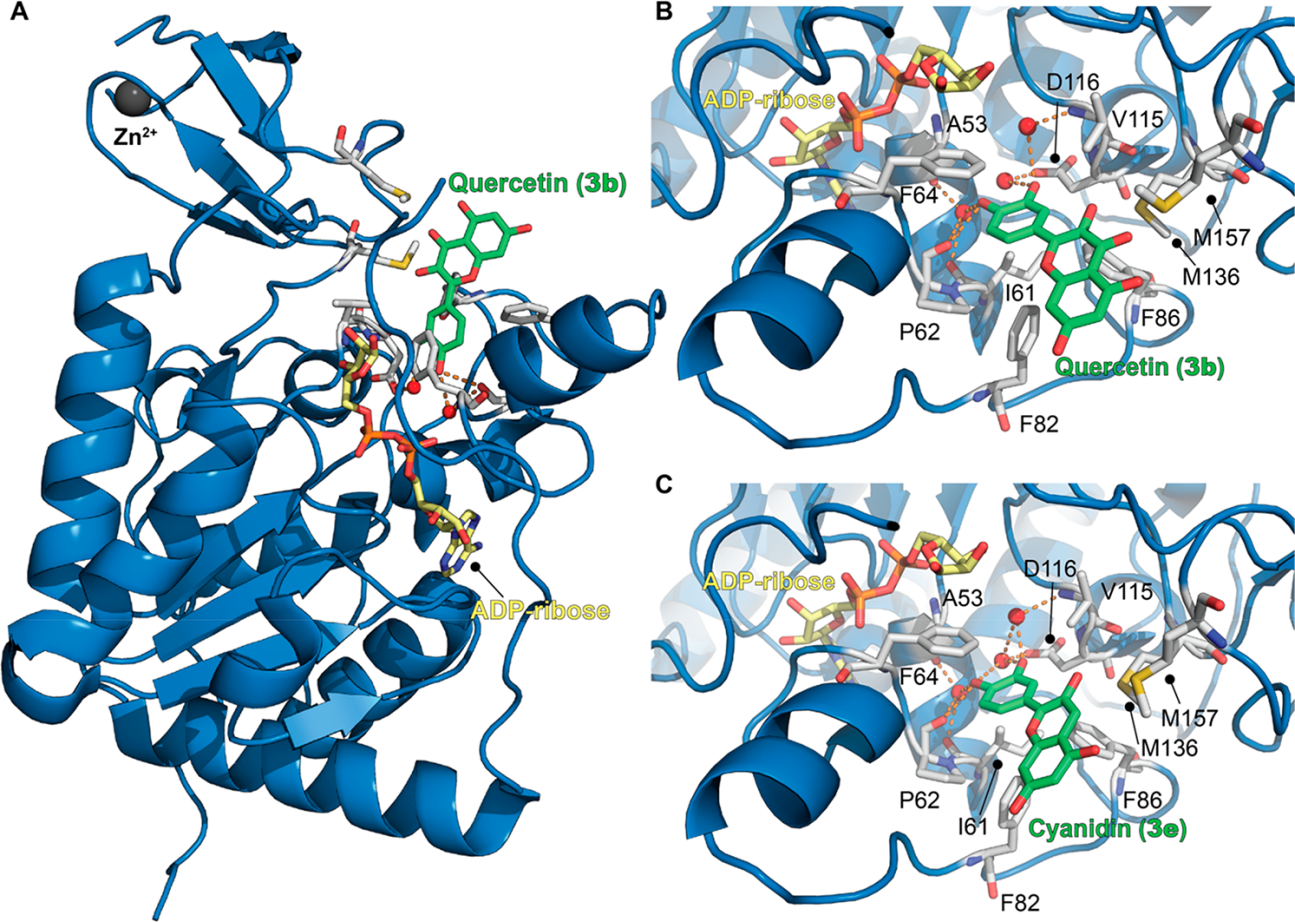
(A) Structure of SIRT6 in complex with ADP–ribose (yellow) and quercetin (**3b**) (green) (PDB ID: 6QCD). (B) Focus on **3b** binding site showing the presence of key water molecules (red spheres) mediating protein-compound interaction. (C) Structure of SIRT6 in complex with ADP–ribose (yellow) and cyanidin (**3e**) (green) with a focus on the **3e** binding site (PDB ID: 6QCH). Key residues for compounds’ binding are labeled, and polar interactions are shown as dashed orange lines.

Nonetheless, flavonoids could represent useful hit compounds for the development of SIRT6 activators thanks to the release of SIRT6–**3b** and SIRT6–**3e** cocrystal structures (PDB IDs: 6QCD and 6QCH, respectively). These structures indicated that **3b** and **3e** interact with SIRT6 at the distal end of the hydrophobic acyl-binding pocket, with surface contacts with the β6/α6 loop that caps this channel (Figure 6).^104^ In both cases, the catechol portion (ring B) is inserted in the acyl-binding pocket with the 4′-hydroxyl group forming a hydrogen bond with Pro62 backbone oxygen and with a conserved water molecule that in turn forms a hydrogen bond with the backbone oxygens of Ala53 and Ile61. Similarly, the 3′-hydroxyls of both molecules are hydrogen-bonded with another conserved water molecule that is in contact with the side-chain oxygen of Asp116. In the case of **3b**, the chromen-4-one moiety (rings A and C) forms hydrophobic contacts with Phe64/82/86, Val70/115, and Met136/157 (Figure 6B). As for **3e**, although the density of this portion was weaker, it was positioned in the same area as **3b**, suggesting a similar binding mode.^104^ Compared to **3b**, **3e** lacks the carbonyl group pointing toward Met136/157, which may be one of the reasons for its higher potency. Indeed, this group may represent a steric hindrance, and its absence allows optimal hydrophobic contacts between ring C and Met136/157 (Figure 6C).

The crystal structures of SIRT6 in complex with **3b** and **3e** enable the identification of key features for ligand binding and, likely, could be exploited to develop new compounds containing only the hydroxyl groups essential for the interaction with the target, thus decreasing the polyphenolic character. Moreover, computational scaffold hopping^120,121^ approaches integrated with AI-driven drug discovery^122^ could allow the design of derivatives bearing a different core but retaining the moieties important for SIRT6 interaction. Overall, these strategies could enable molecules with increased specificity and potency to be obtained.

Another naturally occurring molecule showing SIRT6 activation is fucoidan (**4**), a heterogeneous sulfated polysaccharide present in brown algae. Its backbone consists of repeating (1 → 3) or (1 → 3) and (1 → 4) linked α-l-fucopyranose residues, in which some hydroxyl groups form sulfated esters (Figure 5, lower panel).^123^ The oversulfated fucoidan subtype extracted from *Fucus vesiculosus* displayed a 355-fold increase of SIRT6 activity at a 100 μg/mL concentration. In addition, when tested against other SIRTs (SIRT1/2/3), it did not display significant changes in activity, suggesting a specific action toward SIRT6. **4** was also able to activate SIRT6 acetylation toward H3K9 *in vitro*. According to the authors of the study, sulfate esters may play a central role in SIRT6–**4** interaction and hence SIRT6 activation.^123^ However, the heterogeneity of the mixture, the polymeric nature of the compound, and the absence of kinetic data makes it difficult to compare this macromolecule to small molecules and to devise structure–activity relationships.

**Figure 7:**
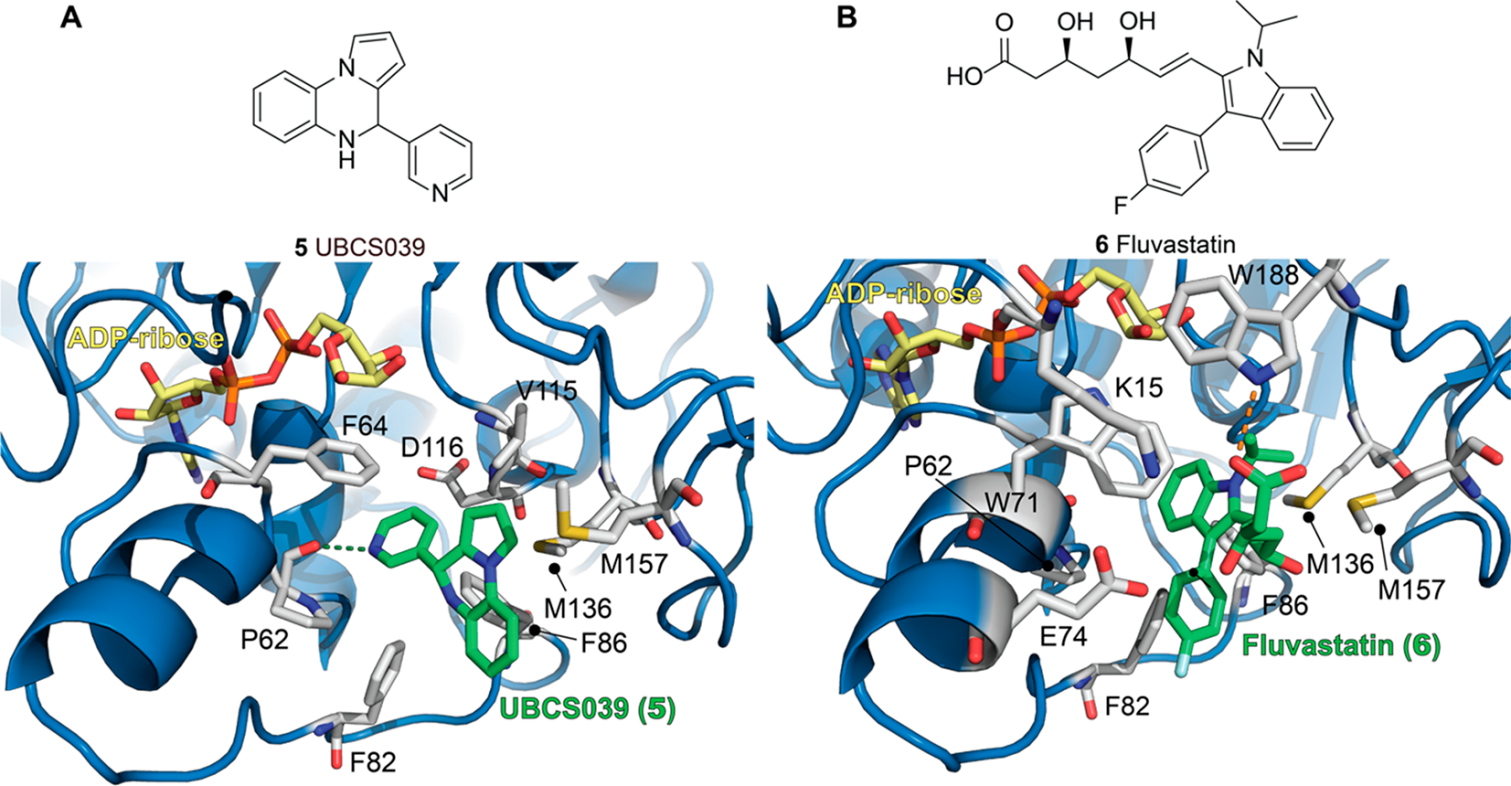
(A) Upper panel: Molecular structure of UBCS039 (**5**). Lower panel: Structure of SIRT6 in complex with ADP–ribose (yellow) and **5** (green) with a focus on the **5** binding site (PDB ID: 5MF6). (B) Upper panel: Molecular structure of fluvastatin (**6**). Structure of SIRT6 in complex with ADP–ribose (yellow) and **6** (green) with a focus on the **6** binding site (PDB ID: 6ZU4). Key residues for compounds’ binding are labeled, and polar interactions are shown as dashed orange lines.

The first synthetic SIRT6 activator is the pyrrolo[1,2-*a*]quinoxaline derivative UBCS039 (**5**, Figure 7a, upper panel), which exhibited an EC_50_ of 38 μM and 3.5 maximum activation of SIRT6 in H3K9Ac peptide deacetylation assays.^124^**5** showed specific binding on SIRT6, with no significant effects on basal SIRT1, 2, and 3 deacetylation activities. Notably, it stimulated SIRT5 desuccinylation activity (2-fold increase at 100 μM), the physiologically dominant activity of this enzyme. The **5**-SIRT6 cocrystal (solved at 1.87 Å resolution, PDB ID: 5MF6) indicated a similar binding mode to **3b** and **3e**, with the compound occupying the exit of the acyl channel pocket and exposing the benzene moiety of the quinoxaline to solvent. The tricyclic portion of the molecule likely forms a methionine–aromatic ring interaction with Met136 along with weak hydrophobic interactions with Trp71, Phe82, Phe86, Ile185, and Met157. In addition, the pyridine nitrogen forms a hydrogen bond with the backbone carbonyl of Pro62 (Figure 7a, lower panel); this interaction represents a key anchoring point as a shift of the position of the nitrogen led to decreased SIRT6 affinity and activation. Comparison of this crystal structure with the cocrystal of SIRT6 and myristoylated peptide indicates that UBCS039 overlaps with the last seven carbons of the myristoyl chain. In addition, comparison with the SIRT6/ADP–ribose/**3b** cocrystal indicates that the compounds share a similar binding site. The **5** pyridine portion overlaps with **3b** cathechol moiety, and both engage in the key interaction with Pro62. In addition, the pyrrolo[1,2-*a*]quinoxaline moiety of **5** and the chromen-4-one are involved in similar hydrophobic interactions. One difference relies on the fact that **3b** possesses a carbonyl group pointing toward Met136/157, which may impair optimal hydrophobic contacts between the aromatic ring and the methionine residues. Differently, the **5** tricyclic system is positioned in a privileged location for aromatic and hydrophobic interactions with Met136/157, thus explaining its higher potency compared to **3b**. Although **5** did not display significant inhibition of SIRT6-mediated demyristoylation, as the binding affinity for the myristoylated peptide is much higher, addition of myristoylated peptide decreased **5** binding by an order of magnitude, thus indicating competition for the same binding site. Compound **5** was also tested using physiological substrates, such as full-length histones extracted from calf thymus and HeLa nucleosomes. In both cases, Western blot analysis indicated H3K18 deacetylation in the presence of **5**.^124^ Follow-up studies indicated that **5** causes SIRT6 activation in a different subset of cancer cell lines, including NSCLC, colon and epithelial cervix carcinoma, and fibrosarcoma. **5**-mediated SIRT6 activation led to decreased H3K9 and H3K56 acetylation and autophagy-related cell death.^125^ This study represents the first evidence of in-cell small-molecule-mediated SIRT6 activation, suggesting a potential therapeutic exploitation of this activity.

A compound screening for drug repurposing recently identified the HMG-CoA reductase inhibitor fluvastatin (**6**, Figure 7B, upper panel), already approved for hypercholesterolemia treatment, as a SIRT6 activator.^126^**6** showed an EC_50_ = 7.1 μM and decreased H3K9 and H3K56 acetylation in HepG2 cell lines. This effect was accompanied by increased nuclear translocation of SIRT6. In addition, **6** treatment increased levels of phosphorylated AMPKα, which in turn promoted SREBP1 phosphorylation at Ser372. In addition, cleaved SREBP1 was negatively regulated. These results are in line with previous reports suggesting that SIRT6 overexpression represses SREBP1/2 through the AMPK pathway.^99^ Interestingly, a subsequent study found a much higher EC_50_ (>250 μM) for **6**-mediated SIRT6 activation, though it could reach 3.5-fold maximum activation at 1 mM.^127^ Compound **6** also displayed weak inhibition of SIRT1/2/3, while it did not affect SIRT5 activity. Nonetheless, the authors managed to cocrystallize **6** with the *N*-terminally truncated SIRT6 (13–308) and ADP–ribose and solved the structure at 2.46 Å (PDB ID: 6ZU4, Figure 7B, lower panel). **6** interacts with SIRT6 at the exit of its acyl channel in its acid form, rather than lactone, forming a hydrogen bond with Trp188 through its carboxyl group. In addition, the fluorophenyl and isopropyl residues point toward the channel exit, while the heptenoic acid moiety interacts with a surface formed by Lys15, Trp71, and Glu74. The indole moiety has a similar positioning of the pyridine ring of **5**,^124^ as it is oriented toward the hydrophobic pocket formed by Phe64/82/86, Ile61, Pro62, and Met136. However, bulky substituents, such as the isopropyl and fluorophenyl groups, obstruct the entrance in the pocket, thereby impairing the key polar interactions with the backbone oxygen of Pro62 seen with **5** and other ligands. In summary, the authors of this study suggest that the initially measured low EC_50_^126^ may be a result of an assay artifact and that the reported cellular effects may be due to an indirect action of **6**.^127^ Nevertheless, the elucidation of the **6** binding mode aids the development of modulators possessing the same core scaffold, but different substituents, in order to maximize polar interactions.

**Figure 8:**
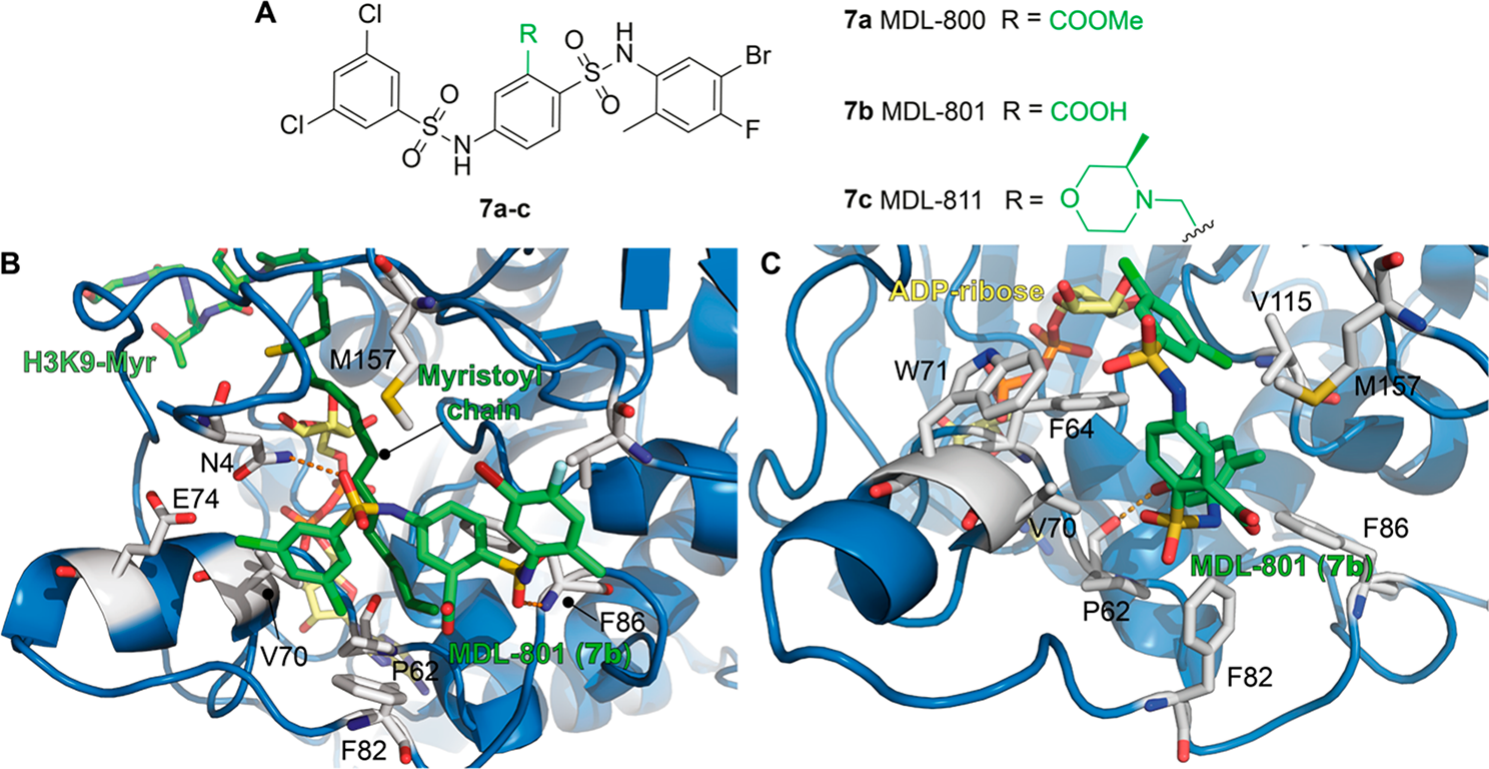
(A) Molecular structures of MDL compounds (**7a**–**c**). (B) Structure of SIRT6 in complex with ADP–ribose (yellow), H3K9-Myr (myristoyl chain in dark green), and MDL-801 (**7b**) (green) with a focus on the **7b** binding site, as reported by Huang et al. (PDB ID: 5Y2F). (C) Structure of SIRT6 in complex with ADP–ribose (yellow) as well as **7b** (green) with a focus on the **7b** binding site, as reported by You and Steegborn (PDB ID: 6XVG). Key residues for compounds’ binding are labeled, and polar interactions are shown as dashed orange lines.

Virtual screening followed by *in vitro* evaluation led to the discovery of a new selective and cellularly active SIRT6 activator, the *N*-phenyl-4-(phenylsulfonamido)benzenesulfonamide derivative MDL-800 (**7a**, Figure 8A).^128^**7a** displayed an EC_50_ value of 10.3 μM, enhancing SIRT6 activity by more than 22 times (at 100 μM), using a synthetic acetylated peptide (RHKK-ac-AMC) as a substrate. When tested on SIRT6 using the H3K9Ac peptide (KQTARK-ac-STGGWW), **7a** exhibited 18-fold maximal SIRT6 activation. In addition, **7a** increased the deacetylation of H3K9 and H3K56 on HeLa-extracted nucleosome substrates in a dose-dependent manner. **7a** did not display any effect on the enzymatic activities of SIRT1/3/4 and HDAC1–11 at concentrations up to 50 or 100 μM. It displayed weak inhibition of SIRT2 (IC_50_ = 100.4 μM) and weak activation of SIRT5 (IC_50_ = 104.6 μM) and SIRT7 (IC_50_ = 187.1 μM). Since the IC_50_/EC_50_ values are 10 times (or more) greater than SIRT6 EC_50_, the compound is considered selective. The analogue MDL-801 (**7b**), in which the methyl carboxylate ester in position 2 of the central benzenesulfonamide ring is replaced by a carboxylic group (Figure 8A), exhibited overlapping SIRT6 activation features with an EC_50_ = 5.7 μM. However, while **7a** was highly cell permeable and accumulated in cells, **7b** had poor cellular permeability and a high efflux ratio. Therefore, the only compound tested for cellular activity was **7a**. This molecule caused a dose-dependent decrease of H3K9Ac and H3K56Ac in HCC cells (specifically Bel7405, PLC/PRF/5, and Bel7402 cell lines), leading to inhibition of their proliferation through SIRT6-mediated cell cycle arrest. In particular, the observed IC_50_ for cell growth (IC_50-growth_) was between 18.6 and 24 μM, depending on the cell line. These results were confirmed in mouse xenograft models, where **7a** suppressed HCC tumor growth through SIRT6 activation. A recent investigation indicated that **7a** inhibits the proliferation of 12 NSCLC cell lines in a dose-dependent manner and caused cell cycle arrest at the G_0_/G_1_ phase in NSCLC HCC827 and PC9 cells, consistent with studies indicating the role of SIRT6 in cell cycle regulation.^16,70^ Notably, it exhibited synergistic activity with epidermal growth factor receptor tyrosine kinase inhibitors (EGFR-TKIs) in osimertinib-resistant HCC827 and PC9 cells and in patient-derived primary tumor cells. Moreover, **7a** suppressed tumor growth in HCC827 cell-derived xenograft nude mice and caused H3 deacetylation and downregulation of p-MEK and p-ERK in tumor tissues.^129^

Huang et al. solved the cocrystal structure of the complex formed by SIRT6, ADP–ribose, H3K9 myristoylated peptide, and **7b** (PDB ID: 5Y2F, Figure 8B).^128^ Given the structural similarities between **7b** and **7a**, the observed features are likely shared between the two compounds. Interestingly, **7b** appeared to interact with SIRT6 in a unique pocket, distinct from the binding site of **3b**, **3e**, **5**, and **6** located in the acyl-binding hydrophobic channel. Indeed, **7b** was shown to interact with a surface-exposed distal region defined by the *N*-terminal residues 1–7, Val70, Glu74, Phe82, Phe86, Val153, and Met157. The 3,5-dichlorobenzene moiety of **7b** is involved in weak polar interactions with Asn40, Val70, and Glu74 and engages π-stacking interactions with Phe82 (Figure 8B). The central 2-carboxybenzenesulfonamide ring is also involved in π-stacking interactions with Phe86, whose importance was confirmed by single-residue mutation experiments showing decreased potency of both **7a** and **7b** toward SIRT6-F86A.^128^ However, a recent report from You and Steegborn argued that the observed electron density could be attributed to a molecule of morpholinoethanesulfonic acid (MES), used as crystallization buffer, rather than **7b**.^130^ Therefore, they determined new crystal structures for SIRT6 in complex with **7b**. They solved the cocrystal of *N*-terminal truncated SIRT6_13–308_ in complex with ADP–ribose and **7b** (PDB ID: 6XV1) as well as in the absence of **7b** (PDB ID: 6XUY). Similarly, they solved the structure for SIRT6_3–308_ (comprising the *N*-terminus) in complex with ADP–ribose and **7b** (PDB ID: 6XVG, Figure 8C), along with a reference structure without the activator (PDB ID: 6XV6).^130^ These structures indicate a different binding mode for **7b**, which does not bind at the distal end of the acyl-binding hydrophobic channel but in the same region as the previously described activators **3b**, **3e**, and **5**. In both SIRT6_13–308_–**7b** and SIRT6_3–308_–**7b** structures, the activator engages in extensive hydrophobic interactions, the central 2-carboxybenzenesulfonamide is packed between Val70, Trp71, and Met157, and the 5-bromo-4-fluoro-2-methylaniline portion interacts with Phe64, Val70, Phe82, Phe86, and Val115. In addition, bromine forms a halogen bond with the backbone amide oxygen of Pro62, which has been shown to be a key residue for small-molecule interactions with SIRT6 (Figure 8C). Notably, the interaction with Pro62 is missing in the binding mode illustrated by Huang et al.^128^ The 3,5-dichlorobenzene moiety is less defined and seems to be largely solvent-exposed. Hence, the structures from the two groups display rather different binding modes for **7b**, whose orientations within SIRT6 are perpendicular to each other in the two studies. In response to this report, Huang et al. crystallized SIRT6 with and without **7b** using the same conditions as in their original publication (PDB ID: 7CL0 for SIRT6 in complex with ADP–ribose and H3K9 myristoyl peptide; PDB ID: 7CL1 for SIRT6 in complex with ADP–ribose, H3K9 myristoyl peptide, and MDL-801).^128,131^ They showed that, in the absence of **7b**, the buffer molecule MES does not fit properly the originally proposed ligand-binding pocket. In addition, the newly solved cocrystal in the presence of **7b**, although possessing lower overall resolution (3.2 Å for 7CL1 vs 2.53 Å for 5Y2F), has better electron density for the activator and confirms their initial findings.^131^ Importantly, Huang et al. crystallized SIRT6 in the presence of the H3K9 myristoyl peptide, which is absent in the crystallization mixture of You and Steegborn. Overall, the observed differences in the **7b** binding mode may be ascribed to the presence of the substrate, which influences the interaction between the small molecule and SIRT6. Indeed, the crystal structure represents just a conformational state of the protein, whose conformational dynamics can be altered by the presence of ligands, thereby leading to alteration of key interactions between a small molecule and their target. Hence, structures of SIRT6–**7b** from both groups may be equally valid and represent two different states of the protein, regulated by the presence of substrate. Nonetheless, further experiments, including the structure of **7b** in the presence of acetylated substrate may help to further clarify this controversy.

The replacement of the methyl carboxylate with an *N*-methyl-3-methylmorpholine at the C3 position of the central benzene ring of **7a** led to compound MDL-811 (**7c**, Figure 8AA) with improved activity (EC_50_ = 5.7 μM) and bioavailability in C57BL/6J mice (*F*%_MDL-800_ = 71.33% vs *F*%_MDL-811_ = 92.96%).^132^ The improved activity may be explained by the higher number of interactions that the *N*-methyl-3-methylmorpholine moiety can establish. Indeed, it presents an exposed oxygen that can act as a hydrogen bond acceptor and a methyl group potentially involved in hydrophobic interactions. The compound enhanced deacetylation of H3K9, H3K18, and H3K56 in nucleosomes extracted from HeLa cells as well as in HEK293T cells in a dose-dependent manner. **7c** was shown to be specific for SIRT6, as it was not able to affect the activity of SIRT1–3, SIRT5, SIRT7, and HDAC1–11 at concentrations up to 100 μM. According to docking studies, the 3-methylmorpholine moiety may participate in hydrophobic interactions with Phe82, Thr85, and Phe86 of SIRT6, and the oxygen can form a hydrogen bond with the backbone amide of Phe86. As already mentioned, CRC is a type of cancer characterized by heavy downregulation of SIRT6.^8^ Notably, **7c** displayed dose-dependent reduction of H3K9Ac, H3K18Ac, and H3K56Ac levels in different CRC cell lines and antiproliferative effects associated with marked G_0_/G_1_ cell cycle arrest. In line with this, **7c** suppressed CRC growth in patient-derived organoids and showed antitumor efficacy in cell-line-derived and patient-derived xenograft (CDX and PDX, respectively) models as well as in a spontaneous CRC mouse model. Mechanistically, cytochrome P450 family 24 subfamily A member 1 (CYP24A1), which had been previously shown to be aberrantly overexpressed in CRC,^133,134^ was identified as a new target gene of SIRT6. **7c** also exhibited synergistic activity with vitamin D_3_ in suppressing CRC proliferation. Interestingly, vitamin D_3_ is both a substrate and transcriptional regulator of CYP24A1 and has shown antitumor efficacy in CRC.^135,136^

**Figure 9:**
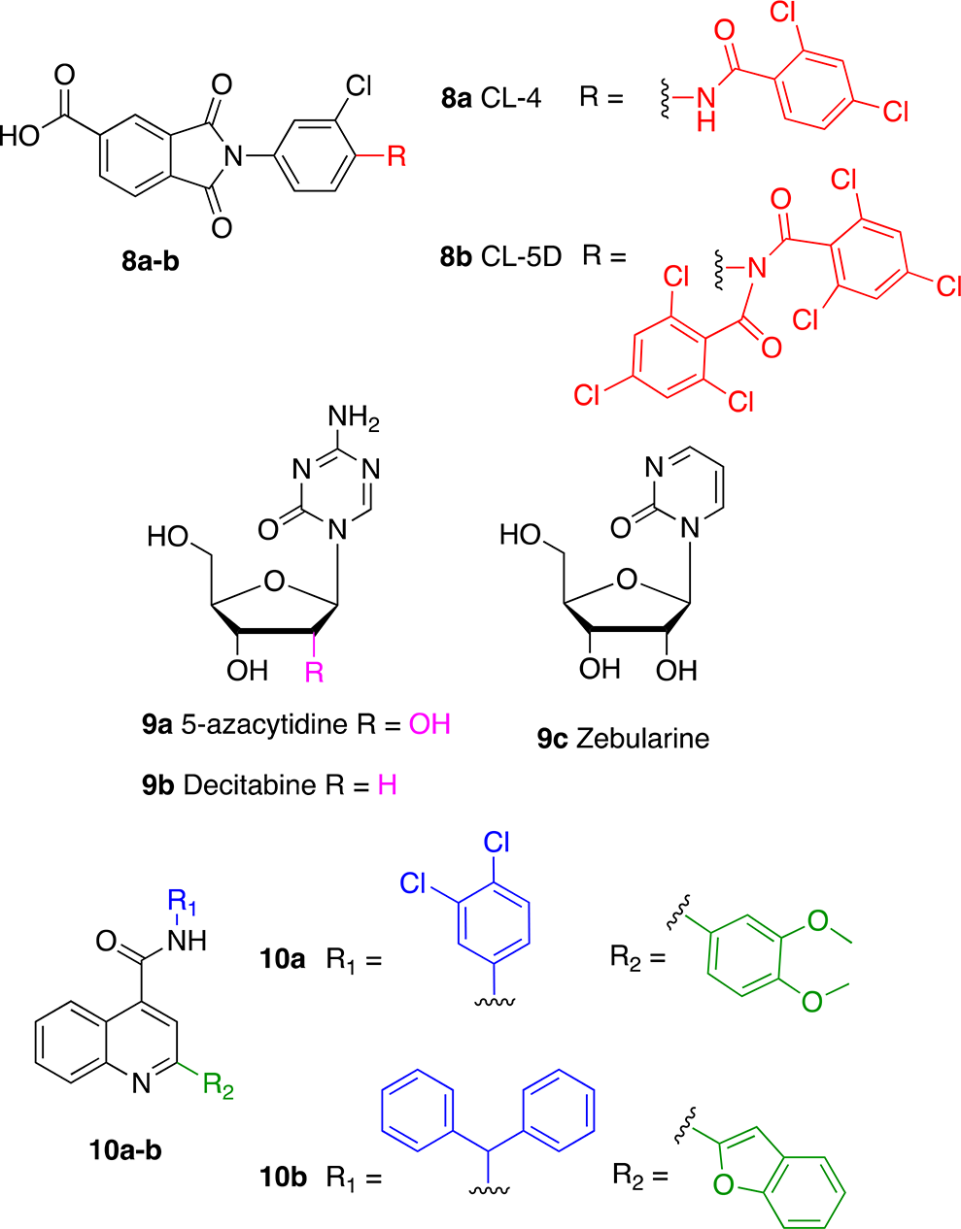
Further synthetic SIRT6 activators.

Activity-based screening of lipid-like molecules led to the discovery and optimization of CL4 (**8a**), a compound consisting of a 4-carboxyphthalimide conjugated to an *N*-(2-chlorophenyl)2,5-dichlorobenzamide. **8a** displayed an EC_50_ = 97 μM and 17 maximum-fold activation of SIRT6 deacetylase activity. In addition, it displayed selectivity over SIRT1–3 and SIRT5.^21^ Therefore, **8a** represented an ideal lead compound for the development of selective SIRT6 activators. Removal of chlorine atoms from either the 2,5-dichlorophenyl moiety or both benzene rings led to the suppression of SIRT6 activation, while progressive addition of chlorine restored this ability (Figure 9, upper panel). Finally, addition of a double-trichlorobenzoyl group at the aniline nitrogen led to CL5D (**8b**), which showed 7-fold increased potency over **8a**, with an EC_50_ = 15.5 μM. Notably, the methyl ester of **8b** did not show any activity. The data obtained from the development of **8b** indicate that electron-withdrawing groups on the aromatic rings are crucial for SIRT6 activation in this series of molecules. In addition, the anionic headgroup (the carboxylic acid) is also essential for activity, and it is probably involved in hydrogen bond interactions. The maximum-fold activation of **8b** was measured in terms of the *k*_cat_/*K*_m_ ratio, which was ∼50 under steady-state conditions. **8b** displayed competitive inhibition of demyristoylation (*K*_i_ = 13.4 μM), suggesting occupation of the acyl-binding pocket, although structural data are missing. **8b** also stimulated SIRT6 deacetylase activity in a time-dependent fashion in full-length histones extracted from HEK293T cells.^3^

A recent study that evaluated the influence of the FDA-approved DNA hypomethylating agents (DHAs) on sirtuin family members showed that the nucleoside analogues 5-azacytidine (5AC, **9a**), decitabine (DAC, **9b**), and zebularine (**9c**) increase SIRT6 enzymatic activity (Figure 9, middle panel).^137^**9a** and **9b** increased SIRT6 activity after 12, 24, and 48 h of incubation at 0.25 and 0.5 μM; albeit, no dose-dependency was observed. Moreover, while the maximum activation (1.3-fold activation) for **9a** was observed after 48 h, **9b** exhibited 1.5-fold activation after 12 h, followed by a decrease in activation efficiency at 24 and 48 h. **9c** could also activate SIRT6 deacetylase activity, although at higher concentrations (0.5 and 1 μM), with 1.4 maximum-fold activation observed after 48 h of incubation. In addition, both **9a** and **9c** (but not **9b**) reduced the enzymatic activity of SIRT1, while the activity of SIRT2, SIRT3, and SIRT5 was not affected by any of these compounds. Although these data indicate that these compounds activate SIRT6, the lack of dose dependency suggests that they have been tested far below their EC_50_; hence, the maximum activation values presented here should be taken cautiously. In line with these results, U937 leukemia cells treated with 0.5 μM **9b** for 24 and 48 h displayed decreased levels of H3K9Ac and H3K56Ac, according to Western blot experiments. Further ChIP-Seq analysis of bone marrow cells derived from six AML patients and treated with **9b** indicated changes of H3K9 acetylation at 187 gene loci; specifically, 102 genes displayed an acetylation decrease, while 85 genes showed an acetylation increase. The authors of this study speculated that the unexpected increase in acetylation may be a consequence of differential effects of **9b** on both HATs and HDACs or possible upregulation of the HAT enzymes targeting H3K9. Signaling pathway analysis showed that H3K9 acetylation changes are related to pathways like EGF/EGFR and Wnt/Hedgehog/Notch, which are associated with AML. Although the study lacks details about the connection between SIRT6 inhibition and the overall antitumor effect of **9b**, it highlighted a possible second mechanism of action of nucleoside analogues, which is worth exploring. Indeed, these molecules seem to be active at relatively low concentrations; hence, a complete biochemical evaluation by SAR studies may lead to the development of selective SIRT6 inhibitors.

A virtual screening campaign performed using the SIRT6–**5** (PDB ID: 5MF6)^124^ complex led to the identification of the initial hit **10a** that was further optimized to provide the 2-(1-benzofuran-2-yl)-*N*-(diphenylmethyl) quinoline-4-carboxamide (**10b**) as a potent and selective small-molecule activator of SIRT6 (Figure 9, lower panel).^138^ In docking experiments, **10a** appeared to bind at the distal end of the hydrophobic channel and engage in π–π interactions with Phe86 with its quinoline scaffold as well as σ–π interactions with the amide backbone of Ala7 through the 3,4-dichlorobenzene moiety. When tested *in vitro*, compound **10a** increased SIRT6 activity by 50% (EC_1.5_) at ∼27 μM. From the docking model, it appeared that the space around the 3,4-dichlorobenzene group and 3,4-dimethoxybenzene group was not occupied; hence, modifications were executed to add moieties that would strengthen the interactions between the molecule and SIRT6. This led to compound **10b**, where the 3,4-dichlorobenzene and the 3,4-dimethoxybenzene were replaced by a diphenylmethane group and a 2-benzofuranyl moiety, respectively. Compound **10b** displayed activation toward both SIRT6 deacetylase and deacylase activities, with EC_50_ values of 5.35 and 8.91 μM for deacetylation and demyristoylation, respectively. **10b** showed no influence on the enzymatic activity of SIRT2, SIRT3, SIRT5, and HDAC1–11. It weakly inhibited SIRT1, but the IC_50_ value for SIRT1-mediated deacetylation (IC_50_ = 171 μM) is more than 30 times higher compared to SIRT6 EC_50_, thereby indicating in any case an appreciable selectivity. According to docking experiments, compound **10b** interacts with SIRT6 in a similar way to its parent compound and presents some extra interactions given by the different substituents. Indeed, the benzofuran forms hydrogen bonds with Met157 and Lys160 and a π–σ interaction with the amide group of Thr156; in addition, the *N*-benzhydryl group is inserted in the allosteric pocket and establishes hydrophobic interactions with Tyr5, Val70, Phe82, Pro62, and Pro80, and one of its benzene rings forms π–π interactions with Phe86. According to this model, although located in a similar region, compound **10b** binds SIRT6 more toward the end of the hydrophobic channel compared to **5**, which may justify **10b**-mediated enhancement of SIRT6 demyristoylation activity. Compound **10b** suppressed the proliferation and caused cell cycle arrest in the G2 phase of PANC-1 and BXPC-3 PDAC cell lines. Cellular thermal shift assay (CETSA)^139^ performed in intact cells confirmed that **10b** (at 25 μM concentration) interacts with SIRT6 in cells. In addition, **10b** exhibited antitumor activity in a human pancreatic tumor xenograft mouse model associated with a decrease of H3K9 acetylation levels. A preliminary study in male Sprague-Dawley rats also indicated a promising pharmacokinetic profile, although the bioavailability was only 4%. Although **10b** with its low micromolar EC_50_ values is a promising lead compound, we cannot exclude that the effects observed in cells and *in vivo* are related to interactions with off-target proteins beyond SIRT6. Therefore, further functional and target engagement assays such as mass-spectrometry-based thermal profiling,^140,141^ histone deacetylase assay homogeneous (HDASH) procedures,^142^ fluorescence resonance energy transfer imaging (FRET) probes,^142^ and affinity-based protein profiling (ABPP)^143^ seem necessary to clarify this point. Moreover, genetic studies^144,145^ alone and in combination with compound treatment in both cellular and animal PDAC models would be required to confirm the causal link between the observed phenotypes and SIRT6 activation and to conclusively assess the therapeutic potential of **10b** in this tumor context.

### SIRT6 Inhibitors

Given the double-faced involvement of SIRT6 in cancer and inflammation, inhibition of SIRT6 in specific contexts may represent a successful strategy for cancer management. Indeed, inhibitors may target different SIRT6-mediated pathways that contribute to cancer progression such as DNA repair mechanisms, cell differentiation inhibition, and inflammatory response (Table 2).

**Figure 10:**
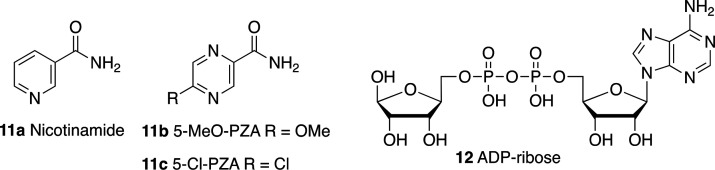
Product-based SIRT6 inhibitors.

Nicotinamide (**11a**, Figure 10) is one of the products of the sirtuin-mediated deacylation reaction and may act as a weak product inhibitor of SIRTs without subclass specificity.^146−148^**11a** has been validated as a SIRT6 deacylation inhibitor through two different assays using H3K9 myristoyl peptides: an HPLC assay yielded an IC_50_ = 153 μM; similarly in a fluorogenic assay, **11a** displayed an IC_50_ = 184 μM.^149^ In a subsequent study, **11a** displayed an IC_50_ for a demyristoylation reaction of 73 μM while showing increased inhibitory potency toward deacylation of H3K9 decanoyl peptide (IC_50_ = 45 μM) and lower potency using H3K9 hexanoyl peptide as a substrate (IC_50_ = 184 μM).^39^

Based on the **11a** analogue pyrazinamide (PZA), Bolivar et al. developed two derivatives with improved SIRT6 inhibition activity (Figure 10): 5-MeO-PZA (**11b**, IC_50_ = 40.4 μM) and 5-Cl-PZA (**11c**, IC_50_ = 33.2 μM). Remarkably, these compounds did not show NAD^+^ competition, hence indicating a different mechanism of action from **11a**.^150^**11c** was reported to be active toward SIRT1, but not SIRT2/3, while **11b** was not evaluated against SIRT1–3. Nonetheless, selectivity against other SIRTs and HDACs need to be ascertained.

ADP–ribose (**12**, Figure 10) also inhibits SIRT6 activity and showed higher potency than **11a** with IC_50_ values of 74 μM (deoctanoylation) and 89 μM (demyristoylation), compared to values of 150 and 120 μM, respectively, for **11a**.^151^

**Figure 11:**
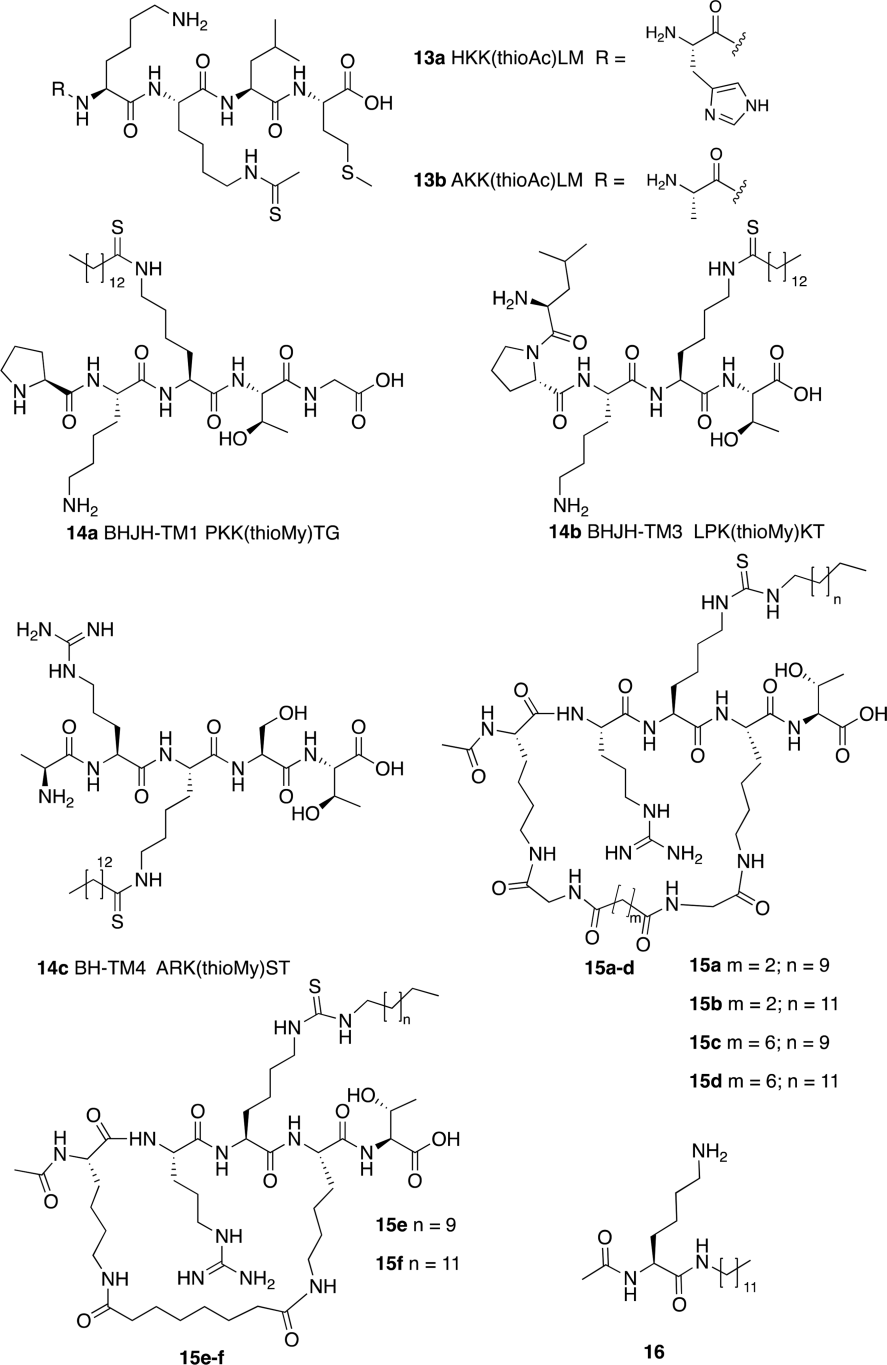
Peptide-based SIRT6 inhibitors.

Another class of inhibitors directly related to the SIRT6 enzymatic mechanism are *N*^ε^-thioacyl lysine peptides, which cause a stall of the catalysis after the nucleophilic attack of the (thio)carbonyl group to the C1′ of nicotinamide-bound ribose that happens in the first step of the catalytic mechanism.^152^ Early reports following the thioacyl peptide strategy let to *N*^ε^-thioacyl lysine pentapeptides **13a** and **13b** (Figure 11, upper panel) showing IC_50_ values toward SIRT6 deacetylase activity of 78 and 47 μM, respectively.^153^ These data indicate that replacement of a His residue with an Ala residue improves inhibitor activity. Both compounds inhibit SIRT1/2 with higher potency compared to SIRT6. Indeed, they both abolish SIRT1/2 almost completely at a 200 μM concentration, while the inhibition of SIRT6 was 62% (**13a**) and 91% (**13b**) at the same concentration. In the case of **13b**, the IC_50_ values for SIRT1 and SIRT2 were 0.38 and 8.5 μM, respectively. These data are in line with the fact that both peptides are based on the sequence of p53, a known substrate of both SIRT1 and SIRT2. Nevertheless, although these molecules are not selective against SIRT6, they represent the first successful example of synthetic SIRT6 inhibitors.

A later study described the development of thiomyristoyl peptides designed on the basis of SIRT6 natural substrates. In particular, compounds BHJH-TM1 (**14a**), BHJH-TM3 (**14b**), and BH-TM4 (**14c**) (Figure 11, middle panel) displayed SIRT6 inhibition in the low micromolar range with IC_50_ values for demyristoylation of 2.8, 8.1, and 1.7 μM, respectively.^154^ These compounds were based on TNFα-K20, TNFα-K19, and H3K9 peptides, respectively. All three peptides were active against SIRT1/2/3, with IC_50_ values between 2.3 and 8.0 μM for all the isoforms, thus indicating a lack of selectivity and a mixed mode of action. Interestingly, they all displayed SIRT6 inhibition and increased TNFα fatty acylation in HEK293T cells with **14b** being the most potent.

More recently, cyclic pentapeptides (**15a**–**f**) harboring a central *N*^ε^-dodecyl- or *N*^ε^-myristoyl-thiocarbamoyl-lysine (Figure 11, middle and lower panels) showed inhibitory activity toward SIRT6 in the nanomolar range (IC_50_ (**15a**) = 256 nM, IC_50_ (**15b**) = 282 nM, IC_50_ (**15c**) = 368 nM, IC_50_ (**15d**) = 319 nM, IC_50_ (**15e**) = 495 nM, IC_50_ (**15f**) = 319 nM). Compounds **15a**–**e** had comparable IC_50_ values for SIRT1, while **15f** an IC_50_ toward SIRT1 2.3 higher compared to SIRT6. Compounds **15e** and **15f**, bearing the same macrocycle bridging unit, were also tested against SIRT2 and SIRT3. Compound **15e** showed moderate selectivity over SIRT2 and SIRT3 (∼2.9-fold and ∼1.5-fold, respectively), while **15f** exhibited high selectivity over the two isoforms (20-fold and 11-fold, respectively). Finally, **15f** was tested against SIRT5, where the results indicated that the molecule is substantially inactive towards this enzyme (IC_50_ > 300 μM). This analysis suggests that the only selective SIRT6 inhibitor is **15f**.^155^ Despite that, **15f** was not able to inhibit SIRT6 inside the human pancreatic cancer BxPC3 cells, likely because of poor cellular permeability given its peptide nature and high molecular weight. Nonetheless, these peptides represent valuable lead compounds for the development of peptidomimetics inhibiting SIRT6.

Recently, Sociali et al. developed a lysine-based compound targeting SIRT6 deacetylase and deacylase activities (**16**, Figure 11, lower panel).^156^ This molecule consists of a lysine residue whereby the *N*^α^-amine group is protected with an acetyl group, while the carboxy group is coupled with a 12-carbon alkyl chain amine. This compound inhibited SIRT6 deacetylation (IC_50_ = 95 μM) without isoform specificity, as it inhibited also SIRT1 and SIRT2 with comparable potency (IC_50_s = 51 and 102 μM, respectively). Remarkably, compound **16** behaved as a deacylation activator showing 52% activation of demyristoylation (EC_50_ = 70 μM) and 80% activation of depalmitoylation at 100 μM while still acting as an inhibitor for SIRT1/2 deacylation (IC_50_ (SIRT1)= 157 μM, IC_50_ (SIRT2)= 177 μM). **16** displayed competitive inhibition toward acetylated peptide, but not NAD^+^, and increased H3K9 acetylation in the MCF-7 breast cancer cell line. Moreover, the activities of key glycolysis enzymes were increased, in line with SIRT6 involvement in downregulation of glycolytic enzymes, and TNF-α secretion was reduced, consistently with the ability of SIRT6 to trigger TNF-α secretion.^22,30^ The results obtained in this study are rather surprising in light of the evidence reported by Feldman et al. that FFAs determine enhancement of deacetylation activity and inhibition of deacylation.^20^ Based on *in silico* data, the authors speculate that the acetyl moiety bound to the Cα amine group may mimic the acetylated substrate, being close to NAD^+^, in agreement with the observed competition with acetylated substrate and not with NAD^+^. However, further experimental evidence is necessary to clarify the binding mode and account for the differential SIRT6 modulation profile.

**Figure 12:**
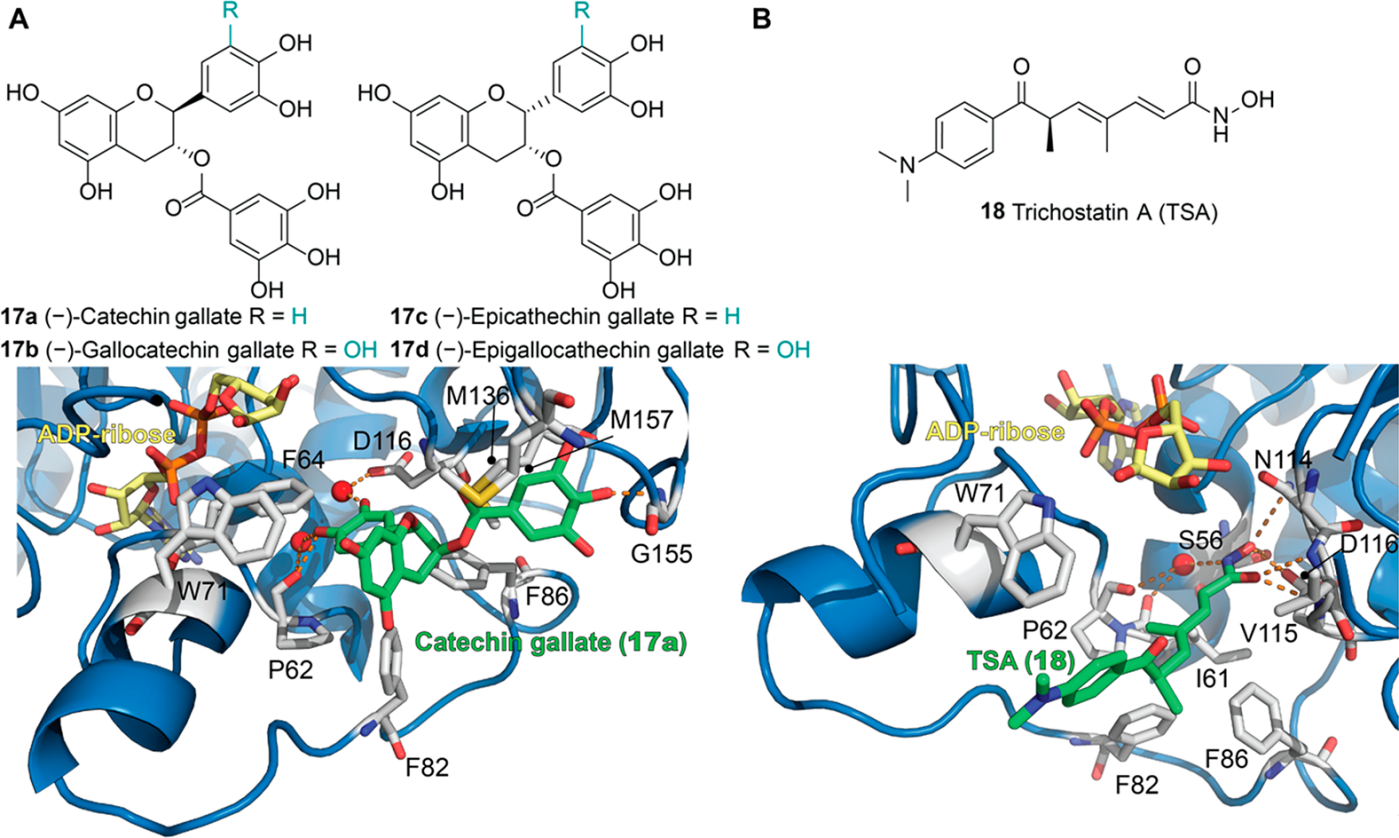
(A) Upper panel: Flavonole-based SIRT6 inhibitors. Lower panel: Structure of SIRT6 in complex with ADP–ribose (yellow) and catechin gallate (**17a**) (green) with a focus on the **17a** binding site (PDB ID: 6QCJ). (B) Upper panel: TSA (**18**) structure. Lower panel: Structure of SIRT6 in complex with ADP–ribose (yellow) and **18** (green) with a focus on the **18** binding site (PDB ID: 6HOY). The binding mode is substantially different from **17a**, although some interactions are shared, such as the key water-mediated hydrogen bond with Pro62 and the hydrophobic contacts with Trp71, Phe82, and Phe86. Key residues for compounds’ binding are labeled, key water molecules for the protein-compound interaction are represented as red spheres, and polar interactions are shown as dashed orange lines.

Interestingly, the **3b** derivatives (−)-catechin gallate (**17a**) and (−)-gallocatechin gallate (**17b**) displayed inhibition of SIRT6-mediated deacetylation in the low micromolar range (IC_50_ (**17a**) = 2.5 μM; IC_50_ (**17b**) = 5.4 μM).^103^ The epimers of compounds **17a** and **17b**, (−)-epicatechin gallate (**17c**) and (−)-epigallocatechin gallate (**17d**), displayed lower activity toward SIRT6, with ∼60 and ∼40% inhibition at 100 μM, respectively, compared to ∼85–90% inhibition of **17a**–**b** at the same concentration. Structurally, these compounds differ from **3b** in ring C, which is reduced and presents a 3,4,5-trihydroxybenzoyl substitution. The **17a**–SIRT6 cocrystal (PDB ID: 6QCJ, Figure 12A) indicated that the inhibitor shares the same binding site as **3b** with identical conformations of the catechol groups, while the chromen-4-one of **17a** was rotated to accommodate the bulky trihydroxybenzoyl moiety. Ring C interacts with Trp71 of the acyl channel exit, and the trihydroxybenzoyl portion forms hydrophobic interactions with the other side of the channel and a hydrogen bond with the backbone of Gly155. It appears that the main difference between **3b**-derived activators and inhibitors consists of the presence of the bulky substituent on ring C and consequent tilted position of the chroman, which is saturated in inhibitors **17a**,**b**. This is supported by the fact that the orientation of the pyrrolo[1,2-*a*]quinoxaline of the SIRT6 activator **5** is similar to the ring C of **3b** derivatives, rather than **17a**. Nonetheless, these compounds were not tested against other SIRTs or HDAC isoforms, so their selectivity needs to be further investigated. In addition, given their polyphenolic structure, both compounds very likely display pleiotropic off-target effects, as previously described for compounds **3a**–**e**. Indeed, **17a**–**b** also inhibit α-glucosidase,^157^ while inhibition of topoisomerases has been widely reported for the **17b** epimer **17d**.^158,159^ This compound also exhibited dual activity toward SIRT3, acting as either an activator or inhibitor depending on the cellular context.^160^ In addition, **17d** inhibits DNMT1^113,114^ and different HAT enzymes such as p300, CBP, PCAF, and Tip60.^86,87^ Even though **17a**–**b** have poor specificity, the availability of the SIRT6–**17a** cocrystal structure could be exploited by medicinal chemists for drug design, as previously mentioned in the case of activators **3b** and **3e**.

Trichostatin A (**18**, TSA), a hydroxamate derivative known for its nanomolar inhibitory activity of class I and II HDACs given its zinc-chelating properties, was recently found to inhibit SIRT6.^161^ Though no IC_50_ was calculated, the *K*_i_ values for **18**-mediated SIRT6 deacetylation were 2.02 μM when using H3K9Ac peptide and 4.62 μM when using p53K382Ac peptide. No inhibitory activity was observed against SIRT1–3 and SIRT5 up to a 50 μM concentration. Kinetic analysis indicated competitive inhibition toward the acetylated peptide but not NAD^+^. The crystal structure of the SIRT6/ADP–ribose/**18** complex (PDB ID: 6HOY, Figure 12B) indicated the binding of **18** to the acyl channel extension of SIRT6, explaining its isoform specificity.^162^ The hydroxamate moiety of **18** engages in polar interactions, including a water-mediated hydrogen bond between **18** nitrogen and the backbone oxygens of Ile 61 and Pro62. The carbonyl group is involved in hydrogen bonds with the backbone nitrogen of Val115 and Asp116. In addition, the **18** hydroxyl moiety acts as a hydrogen bond donor in its interaction with the side chains of Asn114 and Asp116 (Figure 12B). The **18** hydroxamate group mimics **11a** interactions, as confirmed by a competition binding assay in the presence of **11a**. Since no **18**/NAD^+^ competition was observed in activity assays, the authors propose a mechanism whereby **18** interacts with the **11a** binding region following the release of the **11a** moiety from NAD^+^. They also argue that the reported acetylated peptide competition is caused indirectly, by inducing conformational changes leading to clashes with the acylated substrate.

**Figure 13:**
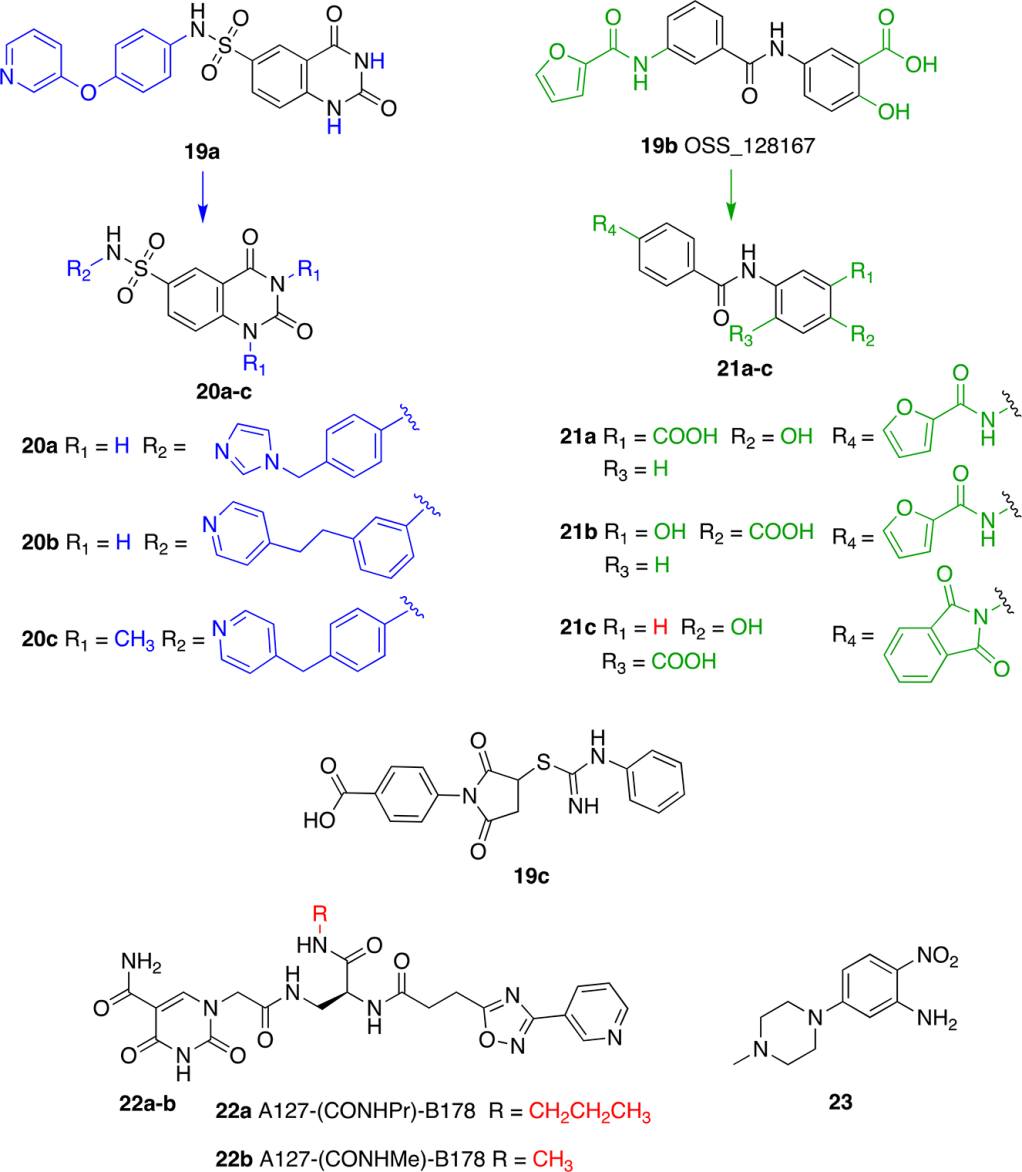
Synthetic small-molecule SIRT6 inhibitors.

Beyond drug repurposing, the first synthetic small-molecule compounds displaying SIRT6 inhibition were identified by Parenti et al. following an *in silico* screening.^163^ This approach led to the discovery of the derivatives **19a**–**c** (Figure 13) possessing IC_50_ values of 106, 89, and 181 μM, respectively. Among them, compounds **19b** (subsequently named OSS_128167) and **19c** displayed selectivity over SIRT1 and SIRT2 (IC_50_ values 8.44 to 19.15 times higher), while **19a** was mildly selective over SIRT1 (IC_50_ = 314 μM) but not over SIRT2 (IC_50_ = 114 μM). The three compounds increased H3K9 acetylation in BxPC3 cells and induced GLUT1 upregulation and consequent augmented glucose uptake in L6 rat myoblasts and BxPC3 cells. This is consistent with reports indicating the role of SIRT6 in GLUT1 downregulation.^59^ Furthermore, the compounds were able to reduce TNF-α secretion. This study shows that small-molecule-mediated SIRT6 inhibition mimics the effects of SIRT6 knockdown. When tested in a murine model of type 2 diabetes, **19a** improved glucose tolerance and reduced plasma levels of insulin, triglycerides, and cholesterol.^164^ Remarkably, **19b** reduced the recruitment of SIRT6 to DNA-damage locations and sensitized primary MM cells, along with melphalan- and doxorubicin-resistant MM cell lines, to DNA-damaging chemotherapeutics.^165^ Compound **19b** also decreased the viability of DLBCL cells, usually displaying SIRT6 overexpression, and inhibited their proliferation in a time- and dose-dependent fashion, through induction of apoptosis and cell cycle arrest at G2/M phase. When tested in a mouse xenograft model with human DLBCL cells, **19b** reduced tumor growth and decreased the levels of the proliferative marker Ki-67.^79^ Nevertheless, these reports lack of target engagement studies demonstrating that **19b** does bind to SIRT6 at least in the cellular context. Hence, the observed *in vivo* phenotype may be a consequence of off-target effects, particularly considering the weak *in vitro* potency of **19b**. To shed light on this, cellular and *in vivo* target engagement studies should be performed.^139−143^ Moreover, the comparison between the phenotypes induced by **19b** treatment, by SIRT6 gene knockdown, and by a combination of the two should also be carried out to clarify the mechanism of action^144,145^ and unambiguously link the observed anticancer effects to SIRT6 inhibition.

Optimization of compound **19a** led to the quinazolinedione derivatives **20a**–**c** (Figure 13, left).^166^ Compounds **20a** and **20b** are characterized by different substituents on the sulphonamide residue; in addition to this, in compound **20c**, the nitrogen atoms of the quinazolinedione core are methylated. These substitutions led to improved SIRT6 inhibition (IC_50_ (**20a**) = 60 μM; IC_50_ (**20b**) = 37 μM; IC_50_ (**20c**) = 49 μM)_._Compound **20a** was slightly selective over SIRT1 and SIRT2 with IC_50_ values of 238 and 159 μM, respectively. Compounds **20b**–**c** exhibited good selectivity over SIRT1 (IC_50_ values were 11 and 133 times higher, respectively) and low selectivity over SIRT2 (2.30-fold and 4.94-fold, respectively), although the activity against other isoforms remains to be tested. It appears that removal of oxygen in the sulphonamide side-chain and the extension of the aliphatic spacer between the aromatic groups (see **20b**) improves the inhibitory efficiency of these derivatives. In addition, the simultaneous oxygen removal from the side chain and methylation of the quinazolinedione nitrogens increases isoform specificity. These derivatives increased H3K9 acetylation in BxPC3, but only compounds **20b** and **20c** caused increased glucose uptake in L6 rat myoblasts and BxPC3 cells. Remarkably, **20a** and **20b** were able to sensitize BxPC3 cells to the chemotherapeutic gemcitabine. Compound **20c** was not evaluated *in vivo*, since it was found to be cytotoxic at a concentration close to its IC_50_ (30 μM). Compounds **19a** and **20b** were found to effectively enhance the anticancer activity of the PARP inhibitor olaparib in Capan-1 cells (a BRCA2-deficient pancreatic cancer cell line). These observations are consistent with previous findings suggesting that SIRT6 knockdown improves the efficacy of chemotherapeutics.^167^

The salicylate derivative **19b** was further optimized yielding the highly selective SIRT6 inhibitors **21a**–**c** (Figure 13, right). Compound **21a** is an analogue of **19b**, in which the furan-2-carboxamide moiety is shifted from 3′ to 4′. Compound **21b** presents the furan-2-carboxamide at the same position as **21a** but has the hydroxyl and carboxylic groups swapped with each other. **21a** and **21b** have IC_50_ values of 34 and 22 μM, respectively. These data indicate that the presence of the furan-2-carboxamide at *para* position massively increases the SIRT6 inhibitory activity, while the swap of hydroxyl and carboxylic groups leads to only a slight improvement of the inhibition. In compound **21c**, a phthalimide moiety replaces the furan-2-carboxamide in 3′, while the carboxylic and hydroxyl groups are in positions 2 and 4, respectively. These modifications furnished a compound with slightly improved inhibitory efficacy, having an IC_50_ of 20 μM.^168^ All compounds displayed selectivity over SIRT1 and SIRT2 (IC_50_ values between 13 and 27 times higher), although the selectivity over other SIRT isoforms needs to be evaluated. Compounds **21a** and **21b** increased H3K9 acetylation and glucose uptake in human peripheral blood mononuclear cells (PBMCs), in line with previous studies and with the roles of SIRT6 in cell homeostasis. Conversely, compound **21c** did not show any effect in cell-based assays, probably due to a lack of cell permeability. Compounds **21a** and **21b** also impaired TNF-α secretion and sensitized pancreatic cancer cells to gemcitabine. Compound **21b** also presented antiproliferative properties in PBMCs.

Compound screening based on a DNA-encoded library designed for NAD^+^-binding pockets led to the identification of two SIRT6 inhibitors with a 5-aminocarbonyl-uracil core (Figure 13, lower panel): A127-(CONHPr)-B178 (**22a**) and A127-(CONHMe)-B178 (**22b**). Both molecules were evaluated in a demyristoylation assay and displayed IC_50_ values of 6.7 and 9.2 μM, respectively.^169^ Compound **22a** was selective over other SIRTs, as inhibition of SIRT1–3, SIRT5, and SIRT7 was less than 10% at 10 μM and was stable in serum after 72 h. It caused an increase of DNA-damage markers and telomere-dysfunction-induced *foci* in primary human umbilical venous endothelial cells (HUVECs), like what was observed following SIRT6 knockdown.^170^ Similarly to other SIRT6 inhibitors, **22a** caused a dose-dependent decrease in the TNF-α levels.

Recently, a series of 1-phenylpiperazine derivatives have been reported as a SIRT6 inhibitors. Among them, 5-(4-methylpiperazin-1-yl)-2-nitroaniline (**23**,Figure 13, lower panel) displayed an IC_50_ of 4.93 μM in a peptide deacetylation assay and showed no activity against SIRT1–3 and HDAC1–11 up to 200 μM concentration.^171^ When tested in BxPC-3 cells, compound **23** augmented the level of both H3K9 and H3K18 acetylation in a dose-dependent manner and increased GLUT1 expression levels. In addition, it reduced the blood glucose content in a mouse model of type 2 diabetes, thus demonstrating promising lead-like properties.

## Conclusions

Mounting evidence supports the critical roles of SIRT6 in multiple processes regulating both physiological and pathological states. Although SIRT6 shares mechanistic features with other SIRTs, it differs from them, as it greatly depends on FFA activation to increase the efficiency of its enzymatic activities.^20,22^ Through its multiple enzymatic activities, SIRT6 finely regulates not only genome maintenance and DNA repair but also stem cell differentiation, metabolism, and aging. The involvement of SIRT6 in these key processes may explain its dual role in cancer. For instance, DNA repair promotion may help evasion from tumorigenic transformation at early phases of cancer. On the other hand, the same mechanism may facilitate cancer progression at later stages or decrease the effectiveness of cytotoxic drug chemotherapy. It is worth noticing that the upregulation of SIRT6 in certain types of cancers^76−79^ may be representative of a compensatory effect rather than the cause itself of tumor initiation and/or progression.^76^

SIRT6 also regulates crucial proteins involved in sugar homeostasis, as it promotes the expression of glycolytic genes,^59,60^ suppressing gluconeogenesis and increasing insulin secretion, hence having a favorable role in diabetes. The downregulation of glycolytic genes also acts as a tumor-suppressor pathway, since it suppresses the Warburg effect.^58^ SIRT6 also regulates fat metabolism by reducing LDL-cholesterol levels^95^ and triglyceride synthesis as well as promoting fatty-acid β-oxidation,^47^ being a key player in obesity prevention. Like the double-faced role in cancer, SIRT6 has contrasting actions in the regulation of inflammation.^80,84,85^

Although the growing knowledge about SIRT6 biology has been uncovering multifaceted functions in human diseases, the discovery of potent and selective SIRT6 modulators is at its infancy. The notion that FFAs increase the deacetylation efficiency of SIRT6 led to the investigation and discovery of the first SIRT6 activators. Initial hit compounds were derived from simple modifications of fatty acids, such as the ethanolamides **2a** and **2b**.^102^ Subsequent synthetic activators overcame issues directly related to the lipidic structure, such as metabolic instability, poor cellular permeability, and low water solubility. Ligand-based drug design efforts led to **5**, the first synthetic activator yielding cellular effects at mid-μM concentrations.^124,125^ This discovery paved the way for the development of further activators, such as **7a**.^128^ Although the binding mode of the analogue **7b** raised some discussion,^130,131^ it is possible that both proposed models are valid in different conditions, and this controversy reminds us that ligand–protein interactions cannot be always recapitulated by a single-crystal structure. In any case, both **7a** and its derivative **7c**(132) inhibited tumor growth in xenograft models, showing SIRT6 activation efficacy *in vivo* for the first time.^129,132^ The recently described activator **10b**,^138^ developed using the **5** binding mode as a model, displayed efficacy at low micromolar concentrations, being an activator of both deacetylation and demyristoylation activities. Remarkably, it also possessed antitumor activity *in vivo*, even though it showed poor water solubility and very low bioavailability. Notably, CETSA measurements demonstrated that **10b** binds to SIRT6 in cells. Nonetheless, further studies are necessary to verify whether the observed phenotypic effects are genuinely related only to SIRT6 activation. Anyhow, **10b** represents a good lead compound that still necessitates a full validation and optimization of the pharmacodynamic and pharmacokinetic properties to be considered as a therapeutic option in the PDAC context.

In the case of inhibitors, the development of substrate-based peptidomimetics led to compounds **15a**–**f** that displayed SIRT6 inhibition in the nanomolar range, although the only compound tested *in vivo* (**15f**) did not show any effect. Nonetheless, given the high potency, these compounds represent optimal starting scaffolds for further developments, first aimed at improving the cellular permeability. Differently, compounds **19b**, **20b**, and **21b**, developed following structure- and ligand-based drug design strategies, were cellularly active, although they inhibited SIRT6 enzymatic activity only in the micromolar range. **19b** also displayed efficacy in a mouse xenograft model of DLBCL; however, additional analyses are necessary to demonstrate a causal correlation between its anticancer activity and SIRT6 inhibition. Therefore, currently, **19b** can be considered only a hit molecule that needs complete validation and, in case, extensive optimization of potency and selectivity.

Innovative approaches relying on high-throughput compound testing hold great potential for drug discovery. Compound **22a** has been discovered by means of DNA-encoded libraries,^169^ a combinatorial approach in which the structure of each molecule is encoded by a conjugated DNA identifier sequence.^172,173^ This method allows quick testing of millions of combinations of fragments using micrograms of protein, offering the exploration of a vast chemical space. The successful application of this approach to SIRT6 led to a low micromolar inhibitor (**22a**) endowed with cellular activity. The application of this technique also to SIRT6 activator discovery would be very interesting.

Finally, compound **23** represents an exciting prospect. It showed low micromolar activity *in vitro* and was able to cause blood glucose reduction in a mouse model of diabetes.^171^ Moreover, its simple structure is amenable of modifications that may lead to more potent derivatives upon a proper structural optimization.

Different challenges have been characterizing the path to the discovery of SIRT6 modulators. These include initial difficulties of properly separating activation and inhibition and the suboptimal efficacy of currently discovered modulators, as explained by the absence of nanomolar activators thus far.

The recent discoveries of *in vivo* active compounds (particularly **7c** and **10b**) bring good hopes for the development of further potent and selective SIRT6 activators. In the case of inhibitors, researchers managed to obtain nanomolar or low micromolar compounds such as **15f**, **22a**, and **23**, plus the mid micromolar inhibitor **19b**, which was active both in cell and *in vivo*, although its SIRT6 target engagement needs to be demonstrated. These molecules cover different chemical classes, ranging from peptidomimetics to small molecules, and some of them represent ideal hit/lead compounds for further development.

Anyway, additional efforts are necessary to improve the potency of the currently available SIRT6 modulators, since usually only nanomolar compounds have concrete chances to progress to preclinical and clinical phases.

To this end, structure-based drug design approaches might be particularly beneficial. Indeed, the availability of the crystal structures of SIRT6 in complex with both activators and inhibitors of polyphenolic nature, such as **3b**, **3e**, and **17a**, enable the identification of key features for target recognition and activity modulation. These cocrystals could be exploited to develop new activating or inhibiting compounds whereby only the important hydroxyl groups are kept while removing the nonessential ones, thus abolishing their pleiotropic effects and increasing their specificity. Moreover, in order to increase potency and selectivity, scaffold hopping^120,121^ approaches could be applied to develop molecules bearing different core chemotypes but retaining the key moieties for SIRT6 interaction. The inspection of the X-ray solved crystal structures of SIRT6 in complex with different modulators that bind in the same pocket with similar binding modes such as the activators **3b** (Figure 6B) and **5** (Figure 7A) could be also leveraged for the structural optimization by combining crucial chemical features. For instance, compound **5** might be modified through the addition of polar groups to the pyridine moiety to form hydrogen bonds with the conserved water molecules bridging to Val115 and Asp116. Moreover, the addition of large hydrophobic groups to the benzene ring could allow further hydrophobic interactions to be established with the pocket formed by Ile61, Phe82 and Phe86.

In conclusion, the available cocrystal structures, along with cutting-edge approaches such as artificial-intelligence-driven drug design and DNA-encoded libraries, have great potential in allowing the evaluation of a more diverse chemical space to obtain molecules possessing drug-like properties to facilitate the discovery of new SIRT6 modulators. To date, the ideal scenario for the initial evaluation of SIRT6 targeting molecules relies on the integration of structural approaches with classic biophysical assays^174^ and modern, label-free methods such as those based on mass spectrometry,^175−177^ to allow reliable assessment of protein–ligand interactions and avoid false positives and negatives that may impair the following steps of a drug discovery campaign.

## Abbreviations Used

5AC: 5-azacytidine
5-hmC: 5-hydroxymethylcytosine
5-mC: 5-methylcytosine
ABPP: affinity-based protein profiling
AMPK: AMP-activated protein kinase
Bax: Bcl-2-associated X protein
BER: base excision repair
CETSA: cellular thermal shift assay
CREB: cAMP response element-binding protein
CRC: colorectal cancer
DAC: decitabine
DDR: DNA-damage repair
DLBCL: diffuse large B-cell lymphoma
DNMT1: DNA methyltransferase 1
DSB: double-strand break
EMT: epithelial–mesenchymal transition
E2F1: E2 transcription factor 1
FFA: free fatty acid
G6PC: glucose-6-phosphatase
FRET: fluorescence resonance energy transfer
GCN5: general control nonderepressible 5
GLUT: glucose transporter
HAT: histone acetyltransferase
HCC: hepatocellular carcinoma
HDASH: histone deacetylase assay homogeneous
HIF-1α: hypoxia-inducible factor 1α
HR: homologous recombination
HSCs: hematopoietic stem cells
IGF: insulin-like growth factor
IGFBP: insulin-like growth factor-binding protein
IL-6: interleukin-6
IL-8: interleukin-8
iPSC: induced pluripotent stem cell
LDH: lactate dehydrogenase
LINE-1: long interspersed element-1
lncRNA: long noncoding RNA
MES: morpholinoethanesulfonic acid
MDM2: mouse double minute 2 homologue
MEFs: mouse embryonic fibroblasts
miRNA: micro-RNA
MM: multiple myeloma
MSCs: mesenchymal stem cells
NHEJ: nonhomologous end-joining
NPC: nasopharyngeal carcinoma
NRF2: nuclear factor erythroid 2-related factor
PAINS: pan assay interference compounds
PARP1: poly(ADP–ribose)polymerase 1
PBMC: peripheral blood mononuclear cell
PCPB2: poly(C)-binding protein 2
PCD: programmed cell death
PCK1: phosphoenolpyruvate carboxykinase
PCSK9: proprotein convertase subtilisin/kexin type 9
PDAC: pancreatic ductal adenocarcinoma
PDK1: pyruvate dehydrogenase kinase-1
PFK1: phosphofructokinase-1
PKM2: pyruvate kinase M2
PTM: post-translational modification
RTE: retrotransposable element
RUNX2: runt-related transcription factor 2
SIRTs: sirtuins
SIRT6: sirtuin 6
SUMO: small ubiquitin-related modifier
SREBP: sterol regulatory element-binding protein
TET: ten-11 translocation methylcytosine dioxygenase
TRF2: telomere repeat binding factor 2
TSA: trichostatin A
UTR: untranslated region
WRN: Werner syndrome ATP-dependent helicase
XIAP: X-linked inhibitor of apoptosis protein
